# Study-Grouped Predictive Toxicology of In Vitro Polystyrene Micro- and Nanoplastic Cytotoxicity: Geometry-Derived Administered-Dose Metrics and a Nanoscale-Restricted Sensitivity Analysis

**DOI:** 10.3390/polym18202575

**Published:** 2026-10-19

**Authors:** Deniz Özkan Vardar, Necati Vardar

**Affiliations:** 1 Department of Pharmacy Services, Vocational School of Health Services, Lokman Hekim University, Ankara 06510, Türkiye; 2 Department of Electrical and Electronics Engineering, Faculty of Engineering and Natural Sciences, KTO Karatay University, Konya 42020, Türkiye; necati.vardar@karatay.edu.tr

**Keywords:** polystyrene, microplastics, nanoplastics, cytotoxicity, predictive toxicology, machine learning, dose metrics, particle geometry, surface properties, study-grouped validation

## Abstract

Polystyrene (PS) micro- and nanoplastics show heterogeneous in vitro cytotoxicity across particle characteristics and exposure conditions. We examined whether geometry-derived administered-dose metrics improve out-of-study prediction of in vitro cytotoxicity and whether ≤100 nm restriction improves performance independently of study composition. From the 2443 literature-derived observations across 98 mammalian in vitro studies, 1701 observations from 82 studies formed the primary mass-concentration dataset and 995 from 60 studies formed the ≤100 nm subset. Random forests were evaluated using nested study-grouped cross-validation with inverse-study-frequency weights. A matched dataset of 141 observations from six studies enabled leave-one-article-out comparisons of mass, particle-number, and geometric surface-area metrics. In the nanoscale subset, observation-level mean absolute error decreased from 15.36 to 14.37 percentage points and R^2^ increased from 0.283 to 0.447, whereas study-balanced mean absolute error did not improve (15.37 versus 15.60). In a composition-controlled 22-study analysis evaluated on identical nanoscale test observations, nanoscale-only training showed no clear advantage. Ridge regression favored mass concentration, while random forest showed a small, uncertain particle-number advantage. Geometry-derived administered-dose metrics offered no consistent model-independent advantage. Study-mean R^2^ (0.095) indicates limited generalizability. Mass concentration remains a practical reference within this evidence base; standardized reporting is needed, and geometry-derived metrics reflect administered, not delivered, dose.

## 1. Introduction

Micro- and nanoscale plastic particles represent a distinctive class of polymeric materials whose biological behaviour is shaped by polymer identity, particle geometry, surface chemistry, dispersion state, and the conditions under which cells encounter them [1,2,3,4]. From a polymer-science perspective, these attributes define important interfacial and transport characteristics that influence how particulate polymers behave in biological media. Polystyrene (PS) is among the most widely used model polymers in experimental micro- and nanoplastic research because particles with controlled dimensions, surface chemistries, and fluorescent labels are commercially available and can be investigated across a wide range of biological systems [5,6,7]. In vitro studies have reported alterations in cellular metabolic activity, membrane integrity, oxidative balance, inflammatory signalling, genomic stability, and cell survival following PS particle exposure [8,9,10,11]. Recent mechanistic synthesis further indicates that PS nanoplastics can engage several partly interconnected cellular stress and death pathways, including apoptosis, ferroptosis, necroptosis, and pyroptosis [12]. Despite this expanding evidence base, cross-study interpretation remains difficult because experimental designs vary substantially in cell model, assay platform, exposure duration, serum conditions, particle characterization, and administered dose.

Dose representation is one of the main sources of this incomparability. In in vitro particle toxicology, exposure is commonly expressed as nominal mass concentration, typically in µg/mL or mg/L [13,14,15]. Although experimentally convenient, mass concentration does not preserve the geometric information that distinguishes particles of different sizes, a limitation long recognized in the broader nanotoxicology literature [16]. At an identical mass concentration, a suspension of smaller spherical particles contains substantially more individual particles and presents a greater total geometric surface area than a suspension composed of larger particles. For particles of constant density, particle number scales approximately with the inverse cube of diameter, whereas geometric surface area per unit mass scales approximately with the inverse of diameter [17,18,19]. Consequently, the same nominal mass concentration can represent markedly different particulate burdens depending on particle size. This distinction is particularly relevant to nanoplastic toxicology because particle size and surface properties influence cellular uptake, membrane interaction, protein adsorption, intracellular trafficking, and biological response [4,20,21,22,23].

Particle-number concentration and geometric surface-area concentration therefore provide alternative dose representations that retain physical information lost in a purely mass-based description [15,17,18,19]. These metrics should not, however, be interpreted as direct measures of delivered cellular dose. The fraction of an administered suspension that reaches the cellular interface additionally depends on agglomeration state, effective density, diffusion, sedimentation, medium composition and viscosity, liquid-column height, exposure duration, and culture geometry [24,25,26,27,28,29]. Accordingly, these geometry-derived metrics describe the nominally administered particle suspension under idealized geometric assumptions; they do not reconstruct particle transport to, deposition at, or uptake by the cell surface. This limitation is especially important in the literature-derived analyses, in which primary diameter, density, dispersion state, and exposure geometry are reported inconsistently. Moreover, primary geometric diameter should not be treated as equivalent to hydrodynamic diameter or the agglomerate size prevailing in the exposure medium.

At the interface of polymer characterization and predictive toxicology, the literature-derived modelling provides a complementary framework for testing whether physicochemical particle descriptors and exposure conditions encode reproducible cytotoxic response patterns across independent experiments. Machine-learning and statistical models can integrate multidimensional experimental observations and test whether recurring exposure–response relationships remain detectable beyond the conditions of individual studies [30,31,32,33]. Recent applications illustrate several directions in which this approach is developing. Meta-analysis combined with machine learning has been used to examine micro- and nanoplastic cytotoxicity in Caco-2 cells [34], physicochemical and exposure descriptors have been incorporated into machine-learning-driven QSAR models of microplastic cytotoxicity [35], and transfer-learning approaches have been proposed to address the limited and fragmented nature of currently available toxicity datasets [36]. Taken together, these approaches motivate an evaluation that tests predictive transportability across entire source studies and isolates the contribution of alternative administered-dose metrics using matched experimental observations. Whether apparent performance gains after nanoscale restriction persist when differences in study composition are controlled remains a further methodological question.

Predictive validity in the literature-derived datasets depends critically on preserving study-level independence. Because individual publications may contribute multiple correlated dose levels, exposure durations, particle formulations, and experimental conditions, random row-wise training–test splits can produce optimistic estimates of performance for previously unseen studies [37,38,39]. The same principle applies to comparisons among dose representations. If mass-, particle-number-, and surface-area-based models are trained on different experimental observations because geometric characterization is selectively missing, apparent differences in predictive performance may reflect changes in dataset composition rather than differences among the dose metrics themselves. A defensible comparison therefore requires matched observations, article-grouped validation, and explicit consideration of uncertainty introduced when geometric dose descriptors are derived from incompletely reported particle properties.

Validation strategy alone, however, does not resolve heterogeneity in the model applicability domain. The operational 1–1000 nm particle-size domain considered in the present study spans a broad physicochemical range [40,41,42]. Within this interval, particles differ substantially in the number represented by a fixed mass, total available surface area, diffusion and sedimentation behaviour, cellular-access mechanisms, and potential internalization pathways [20,21,22,23,24,25,26]. The distinction between particles approaching the upper boundary of this domain and those within the stricter nanoscale range around 100 nm therefore has both definitional and biological implications [40,41]. A model spanning this domain may therefore encounter heterogeneous exposure–response relationships, but any observed improvement after restricting the analysis to ≤100 nm can also reflect changes in the contributing studies. Particle-size restriction and study-composition effects must therefore be distinguished before attributing differences in predictive performance to greater nanoscale coherence.

Against this background, two related but analytically distinct uncertainties remain. First, it is unclear whether replacing nominal mass concentration with geometry-derived particle-number or geometric surface-area concentration provides a reproducible improvement in out-of-study prediction when the alternative dose metrics are evaluated on the same experimental observations. Second, it remains uncertain whether apparent differences in predictive performance following ≤100 nm restriction persist when the contributing studies and held-out nanoscale observations are kept comparable. These questions are important because higher apparent model performance does not necessarily indicate better biological representation when study-level clustering, dataset composition, and applicability domain are not explicitly considered [33,34,35,36,37,38,39].

Here, we developed a literature-derived, study-grouped predictive toxicology framework for mammalian in vitro PS particle cytotoxicity that treats polymer-particle size and geometry as explicit components of the model applicability domain. First, we evaluated cross-study prediction of normalized viability-related responses in a harmonized mass-concentration dataset and examined predictive performance within a prespecified nanoscale-restricted subset (≤100 nm). Second, among experimental observations for which mass concentration, particle-number concentration, and geometric surface-area concentration could all be represented, we performed a matched comparison to evaluate whether geometry-derived administered-dose metrics provided more consistent out-of-study prediction than conventional mass concentration. We additionally conducted a composition-controlled sensitivity analysis comparing broad-domain and nanoscale-only training on identical held-out nanoscale observations from studies contributing both particle-size ranges. All validation procedures were grouped by article so that observations originating from the same publication could not contribute simultaneously to model development and held-out evaluation. By distinguishing particle-size restrictions, study-composition effects, and alternative representations of administered dose, the framework was designed to evaluate the robustness of cross-study predictive relationships without assuming that narrower particle-size domains or geometry-derived metrics necessarily improve prediction.

## 2. Materials and Methods

### 2.1. Evidence Identification, Eligibility, and Study Selection

A systematic evidence-identification strategy was developed to capture in vitro studies reporting viability- or cytotoxicity-related responses to polystyrene (PS) particles. PubMed/MEDLINE, Web of Science Core Collection, and Scopus were searched across all years available through 13 September 2026. No restrictions were applied with respect to language, publication year, document type, subject area, or open-access status. The search strategy was deliberately optimized for sensitivity rather than specificity and comprised three complementary components: a modern PS–nanoplastic branch, a legacy PS latex/bead/microsphere branch, and an abbreviation safety-net branch incorporating terms such as PSNP, PS-NP, and PS-NPL. Exposure terms were combined with viability- and cytotoxicity-related terms, including viability, cell survival, metabolic activity, 3-(4,5-dimethylthiazol-2-yl)-2,5-diphenyltetrazolium bromide (MTT), 3-(4,5-dimethylthiazol-2-yl)-5-(3-carboxymethoxyphenyl)-2-(4-sulfophenyl)-2H-tetrazolium (MTS), 2,3-bis(2-methoxy-4-nitro-5-sulfophenyl)-2H-tetrazolium-5-carboxanilide (XTT), water-soluble tetrazolium (WST), Cell Counting Kit-8 (CCK-8), resazurin, neutral red, trypan blue, CellTiter, and live/dead assays. Terms related to machine learning, particle number, or surface area were intentionally excluded so that study retrieval was not conditioned on the analytical framework subsequently applied to the data.

Search documentation followed a Preferred Reporting Items for Systematic Reviews and Meta-Analyses (PRISMA)-style study-flow structure [43], with database-specific search reporting informed by PRISMA-S [44]. Full search strings, branch-specific yields, search dates, and incremental-recall checks were preserved as part of the search audit trail and are provided in the Supplementary Material.

Records retrieved from the three databases were merged into a single bibliographic pool and deduplicated using a prespecified hierarchical procedure. Duplicate records were identified sequentially by exact digital object identifier (DOI) matching, exact PubMed identifier (PMID) matching, and exact normalized-title matching when bibliographic identifiers were compatible and publication years differed by no more than one year. Records with conflicting DOI or PMID information were not merged automatically. Ambiguous or near-matching records were retained for conservative manual review before a final deduplication decision was made.

Studies were considered potentially eligible when they investigated PS-based particles in mammalian cultured cells and reported, or could plausibly contain, quantitative viability-related outcomes, including cell survival or cellular metabolic activity. Because the upper size boundary of nanoplastics remains debated, eligibility was operationally defined as a primary particle diameter of 1–1000 nm [40,41], with a prespecified ≤100 nm subset used to assess robustness under a stricter nanoscale definition. Eligibility was defined according to PS composition and particle size rather than particle origin. Commercially engineered model particles, laboratory-generated or degraded PS particles, and environmentally derived or weathered PS particles were therefore eligible provided that the remaining inclusion criteria were met. Mixed-polymer and mixed-size studies were retained when a PS-specific exposure arm within the target size range could be isolated. Studies were excluded when they were exclusively in vivo, used non-mammalian biological models, investigated only non-PS materials, used PS solely as a culture substrate, scaffold, carrier, tracer, synthesis support, or procedural reference, lacked a separable PS-only exposure arm, or contained no compatible quantitative viability-related endpoint.

Title and abstract screening were performed independently by two reviewers using prespecified Include, Exclude, and Unclear categories. Reviewers completed their assessments without access to the other reviewer’s decisions. Exclusion at this stage required clear evidence of ineligibility from the title and abstract. Missing particle size, incomplete physicochemical characterization, absence of an explicitly stated viability-related endpoint, or uncertainty regarding the presence of an eligible mammalian in vitro arm was not considered sufficient for exclusion; such records were classified as Unclear and advanced to the full-text retrieval stage. Reviewer calibration was performed using an initial 100-record pilot followed, where necessary, by an independent 50-record confirmation set. Agreement was evaluated using raw percentage agreement and Cohen’s κ [45]. Progression to the main screening phase required a prespecified operational threshold of ≥85% agreement and κ ≥0.60 for the binary Advance-versus-Exclude disposition, with both Include and Unclear classified as Advance. Operational definitions were clarified during calibration without changing the underlying scientific eligibility criteria. Include and Unclear decisions advanced to full-text retrieval and, when reports were available, to eligibility assessment, whereas Exclude decisions did not. Disagreements affecting progression were resolved by consensus or, where necessary, adjudication.

At full-text assessment, eligibility required a mammalian in vitro exposure to PS particles within the defined size range, at least one non-zero exposure condition, and an extractable quantitative viability, survival, or metabolic-activity outcome suitable for normalization to the corresponding untreated control. Membrane-damage endpoints such as lactate dehydrogenase (LDH) release alone were not considered sufficient for eligibility. Mixed exposures were retained only when the PS-alone condition could be evaluated separately. When numerical values were unavailable in tables or text but could be reliably recovered from figures, the study remained eligible and the extraction source was explicitly flagged. Non-English articles were retained; machine-assisted translation could be used where necessary, with critical numerical information independently checked. For the study-flow audit, records advanced to full-text retrieval were distinguished from reports actually retrieved and assessed for eligibility. Reports that remained unavailable at workflow closure were recorded as “full text not retrieved” rather than counted as full-text exclusions. More specific reasons for unsuccessful retrieval were not assigned retrospectively because they were not documented in the archived retrieval records.

### 2.2. Data Extraction, Quality Control, and Outcome Harmonization

Data were extracted at the level of the individual experimental condition rather than the article. Each observation was assigned a unique Observation_ID and linked to an Article_ID and Particle_Arm_ID. Extracted variables included publication year, cell model, tissue of origin, assay, exposure duration, reported dose and dose unit, primary particle diameter, particle morphology, surface functionalization, hydrodynamic diameter, zeta potential, polydispersity index, dispersion procedure, serum concentration, particle source, material density and density provenance, normalized viability-related response, variance reporting, replicate information, and assay-interference information where available.

The continuous outcome used for predictive modelling was the normalized viability- or survival-related response expressed as a percentage of the corresponding untreated control. Metabolic assays, including MTT, MTS, WST, CCK-8, and resazurin, were retained as measures of cellular metabolic activity and were not reinterpreted as direct measurements of viable cell number. Response values exceeding 100% were preserved without truncation. Zero-dose controls were retained in the extraction archive for normalization, experimental matching, and quality-control purposes but were excluded from model fitting and performance evaluation.

Quantitative dose–response results were frequently available only in graphical form rather than as numerical values in article text or tables. Figure digitization therefore constituted a major component of the extraction process rather than an exceptional recovery procedure. Of the 1701 observations included in the final Primary Mass-Concentration Dataset, 1641 (96.5%) were figure-derived, and 60 (3.5%) were directly reported numerically. A similarly high proportion was present in the prespecified ≤100 nm subset, in which 961 of 995 observations (96.6%) were figure-derived. Each observation was explicitly classified as Figure-derived or Directly reported, allowing data-source type to be retained as a model predictor and examined in robustness analyses.

Figure-derived values were obtained using a prespecified quality-control procedure. The highest-resolution version of the relevant main-text or supplementary figure available to the reviewers was used for numerical extraction. Axis scaling, dose labels, exposure durations, experimental-group identities, and figure-panel assignments were cross-checked against the corresponding figure caption, Methods section, and accompanying source text before values were entered into the extraction archive. Numerical precision was restricted to the level supported by graphical resolution rather than reporting visually derived estimates with unsupported decimal precision. When dose sequences, axis labels, or figure captions appeared inconsistent with the methodological description, the discrepancy was adjudicated using the complete source context and documented in the quality-control record rather than resolved solely from the graphical estimate. Error-bar type was retained when explicitly reported, whereas numerical variance magnitudes were left missing when they could not be recovered reliably. Pseudo-control values, visually indeterminate intermediate doses, and unsupported dose or unit conversions were not introduced.

During final adjudication, seven studies for which quantitative outcome extraction had remained unresolved were additionally recovered from supplementary figures. These studies contributed 219 figure-derived observations to the frozen extraction archive. These observations represented a subset of the complete figure-derived evidence base rather than the total number of digitized observations.

As an additional post-review quality-control assessment, a separate human researcher who had not participated in the original extraction independently examined 30 selected figure-derived experimental conditions from ten source articles, with three conditions selected per article. The researcher was blinded to the response values recorded in the original extraction archive. Source figures and accompanying descriptions were examined to verify particle identity, exposure duration, dose, figure-panel assignment, graphical scaling, and the corresponding response value. Conditions that could not be read reliably were documented as unresolved rather than assigned estimated values. Independently recovered values were compared with the archived responses using absolute differences in percentage points, including the mean absolute difference and the proportion of readings differing by no more than two percentage points. One additional targeted quality-control condition was examined separately and was not included in the 30-condition comparison. Discrepancies involving graphical interpretation, experimental-condition assignment, or normalization were flagged for source-level review rather than automatically incorporated into the frozen dataset.

Reporting and methodological quality were evaluated using domains informed by the SciRAPnano in vitro tool v2.0 [46]. Reporting quality and methodological quality were treated as distinct constructs rather than collapsed into a single numerical score. Additional PS-specific quality indicators included particle purity, fluorescent-dye controls, monomer or leachate controls, endotoxin assessment, serum conditions, protein-corona reporting, assay-interference controls, dispersion procedures, and fluorescent labelling. Quality indicators were retained for study characterization and sensitivity analyses and were not combined into an aggregate quality score for predictive modelling. For an additional study-quality sensitivity analysis, the primary dataset was restricted to observations from studies classified as High in both reporting and methodological quality. Following completion of extraction and final adjudication, the master dataset was frozen before construction of the analytical datasets; all subsequent analyses were performed on derivative datasets without modifying the frozen archive.

### 2.3. Dose Representation and Analysis Datasets

Administered mass concentration was used as the primary reference dose metric and harmonized to µg/mL when source units were directly convertible. Administered areal mass dose (µg/cm^2^) was retained separately and was not converted to mass per volume unless the source provided the experimental information required for a defensible conversion. For spherical or quasi-spherical particles with defensible primary-diameter and material-density information, particle-number concentration and total geometric surface-area concentration were calculated as geometry-based transformations of administered mass concentration under idealized particle-shape assumptions [15,17,18,19,27,28,29]. These calculated descriptors represent alternative expressions of administered dose rather than estimates of dose delivered to cells; particle transport and deposition at the cellular interface were not modelled. Hydrodynamic diameter obtained by dynamic light scattering was not substituted for primary geometric diameter because it reflects the dispersed particle or agglomerate state rather than primary particle geometry.

Four analytically distinct datasets were defined. The Primary Mass-Concentration Dataset included model-training-eligible observations with non-zero mass concentration, positive exposure duration, and a quantitative normalized response. The Strict ≤ 100 nm Dataset represented the prespecified nanoscale sensitivity subset of the primary dataset. The Secondary Areal-Dose Dataset contained training-eligible observations reported only in µg/cm^2^ and was analyzed descriptively because the number of observations and contributing studies was insufficient for independent predictive modelling. The Metric-Comparable Dataset was restricted to experimental conditions for which mass concentration, particle-number concentration, and geometric surface-area concentration could all be represented for the identical observation. For an additional post-review sensitivity analysis, studies contributing observations both within and above the ≤100 nm threshold were identified to compare broad-domain and nanoscale-only model training on identical held-out ≤100 nm observations.

### 2.4. Predictor Construction

Continuous predictors for the primary predictive models were log10-transformed administered mass concentration, exposure duration, and primary particle diameter. Primary diameter was not replaced by hydrodynamic size when unavailable; instead, missing primary diameter was represented by a missingness indicator and imputed within training folds. To evaluate the influence of diameter imputation, an additional complete-case sensitivity analysis was performed after excluding observations without a numerical primary particle diameter. Serum percentage was retained when reported. Categorical predictors comprised cell model, species, harmonized tissue group, raw assay, harmonized assay family, surface-functionalization group, harmonized surface-charge group, protein-corona reporting, assay-interference control, data-source type (Directly reported or Figure-derived), and particle-size class. To examine potential redundancy between raw and harmonized assay descriptors and between continuous diameter and particle-size class, a reduced-predictor sensitivity analysis was conducted after removing raw assay and particle-size class while retaining the remaining predictors. Article_ID was used exclusively as a grouping variable and was never supplied to the models as a predictor.

### 2.5. Study-Grouped Predictive Modelling

Predictive modelling was designed to evaluate transportability to previously unseen studies rather than interpolation among experimental conditions originating from the same publication. All resampling procedures therefore grouped observations by Article_ID, preventing observations from the same publication from contributing simultaneously to model development and held-out evaluation.

Because the number of extracted experimental conditions varied substantially among studies, model fitting used inverse-study-frequency sample weights. For article i, containing n_i_ eligible observations, each observation was assigned a fitting weight proportional to 1/n_i_. This weighting scheme ensured that articles contributing large numbers of dose, time, or experimental conditions did not exert proportionally greater influence on model fitting.

An initial benchmark analysis compared a median dummy regressor, Ridge regression, random forest (RF) [47], extremely randomized trees [48], and gradient boosting implemented with CatBoost [49]. Benchmark models used Ridge regression with α = 1.0; random forest and Extra Trees with 300 trees, a minimum leaf size of 3, and a maximum feature fraction of 0.65; and CatBoost with 300 iterations, a depth of 6, a learning rate of 0.05, and RMSE loss. These benchmark hyperparameter settings were distinct from those used in the final nested random-forest tuning procedure described below. Stochastic models used a fixed random seed of 42, and inverse-study-frequency weights were applied to all learned benchmark models. Benchmark performance was evaluated using five-fold GroupKFold out-of-fold predictions, with shuffling enabled and a fixed random seed of 42. Mean absolute error (MAE), expressed in percentage points of normalized response, was the principal prediction-error measure. Root mean square error (RMSE) and the coefficient of determination (R^2^) were reported as complementary observation-level metrics. To reduce the influence of articles contributing large numbers of observations, study-balanced MAE was calculated by first computing MAE separately within each held-out article and then averaging the article-specific errors. Study-mean performance was evaluated after aggregating observed and predicted responses within each Article_ID. Given the heterogeneity across source studies, R^2^ was interpreted alongside absolute prediction errors and study-level performance measures rather than against a universal numerical threshold for acceptable prediction.

The final nonlinear analysis used nested grouped cross-validation to separate hyperparameter selection from performance estimation and reduce optimistic selection bias associated with tuning and evaluating models within the same resampling procedure [50,51]. Five outer GroupKFold partitions were used exclusively for performance estimation, while three inner grouped folds within each outer training partition were used for model selection. Both outer and inner grouped splits were generated with shuffling enabled and a fixed random seed of 42. Article_ID defined group membership at both levels.

Random-forest tuning was deliberately restricted to four prespecified combinations of minimum leaf size and maximum feature fraction: 2/0.50, 3/0.65, 5/0.80, and 8/1.00. Each candidate random forest contained 220 trees and used a fixed random seed of 42. Within each outer training partition, the optimal configuration was selected according to the lowest study-balanced MAE across the inner grouped folds. Continuous predictors were imputed using medians estimated from the corresponding training fold. Categorical predictors were imputed using the most frequent category within the training fold and subsequently one-hot encoded, with previously unseen categories ignored during transformation. All preprocessing, categorical encoding, missing-value handling, hyperparameter selection, and model fitting were therefore confined to training data.

Performance uncertainty for the final nested model was quantified from the outer-fold out-of-fold predictions using 1000 Article_ID-cluster bootstrap replicates [52]. In each bootstrap replicate, articles were sampled with replacement and all corresponding observed–predicted pairs from each selected article were retained together, thereby preserving dependence among observations originating from the same study. This procedure quantified uncertainty in held-out predictive performance and did not involve repeated hyperparameter tuning or refitting of the nested modelling pipeline.

The prespecified nanoscale-restricted analysis repeated the complete nested grouped random-forest procedure after restriction to the ≤100 nm subset. Predictor contribution within this subset was evaluated using held-out permutation importance. Within each outer test partition, each predictor was permuted 10 times and the resulting increase in MAE was calculated relative to the unpermuted model. Permutation effects were averaged within each outer fold and subsequently across the five outer folds. These values were interpreted as measures of contribution to held-out predictive performance rather than evidence of causal biological effects.

All analyses were performed in Python 3.13 using scikit-learn 1.8.0 and CatBoost 1.2.8.

### 2.6. Matched Geometry-Derived Administered-Dose-Metric Comparison

The geometry-derived administered-dose-metric analysis was conducted as a secondary matched comparison. The final Metric-Comparable Dataset comprised 141 experimental observations from six independent studies for which mass concentration, particle-number concentration, and geometric surface-area concentration could all be represented for the same experimental condition. All eligible conversions met the predefined High dose-conversion-confidence criteria.

To ensure that differences in predictive performance reflected dose representation rather than differences in sample composition or covariate structure, all three dose-metric models were fitted to exactly the same observations and used identical contextual predictors. Each specification included one log-transformed dose metric—mass concentration, particle-number concentration, or geometric surface-area concentration—together with log10 exposure duration, species, tissue group, assay family, surface-functionalization group, surface-charge group, and data-source type. Primary particle diameter was not entered as an additional predictor in any of the matched specifications because diameter is mathematically embedded in geometry-derived particle-number and geometric surface-area metrics; including it separately would therefore have made the competing model specifications structurally non-equivalent. Nevertheless, excluding diameter from the mass-based Ridge specification could limit its ability to capture diameter-dependent effects. This possibility was examined in an additional post-review sensitivity analysis.

Cross-study prediction in the matched dataset used leave-one-article-out (LOAO) validation. Ridge-regression regularization strength was selected within each training set using three-fold grouped cross-validation. A conservative random-forest comparator was evaluated under the same LOAO partitions. Study-balanced MAE was used as the primary matched-set metric so that the study contributing the largest number of observations could not dominate the dose-metric comparison. Paired contrasts were defined as MAE(mass) − MAE(alternative), such that positive values favoured the alternative dose metric. Ninety-five percent confidence intervals were estimated using 5000 article-cluster bootstrap replicates [52]. For the post-review Ridge sensitivity analysis, the mass-concentration specification was compared with two extended models: one additionally incorporating log10(primary particle diameter) and another incorporating both log10(primary particle diameter) and its interaction with log10(mass concentration). All three specifications were evaluated using the same 141 observations, six held-out article partitions, and article-grouped inner tuning procedure. These supplementary comparisons were kept separate from the original dose-metric analysis. Study-specific held-out MAEs and paired MAE differences were also summarized for each of the six contributing studies to examine variation in predictive performance across articles.

### 2.7. Sensitivity and Robustness Analyses

The prespecified strict ≤ 100 nm analysis repeated the complete nested grouped random-forest framework. Additional exploratory robustness analyses repeated the grouped random-forest pipeline after restricting the primary dataset to directly reported numerical outcomes and, separately, to observations originating from studies reporting an assay-interference control. These subsets were interpreted cautiously because each restriction altered study composition and substantially reduced the number of independent studies.

Additional post-review sensitivity analyses examined the effects of study composition, missing particle-diameter information, predictor redundancy, and study quality. To distinguish the effect of nanoscale restriction from changes in study composition, articles contributing observations both within and above the ≤100 nm threshold were identified. Models trained on the broader particle-size range and on ≤100 nm observations were then compared using identical held-out ≤100 nm observations under article-grouped validation. The influence of missing primary-diameter values was assessed through a complete-case analysis excluding observations without a numerical primary particle diameter. Predictor redundancy was examined by removing raw assay labels and categorical particle-size class while retaining harmonized assay family and continuous primary diameter. Finally, a quality-restricted sensitivity analysis was performed using observations from studies previously classified as High in both reporting and methodological quality according to the SciRAPnano-informed assessments described in Section 2.2. No composite quality score was constructed. These additional analyses retained study-level separation during model evaluation and were interpreted as robustness assessments rather than direct evidence of improved biological predictability.

The areal-dose and geometry-comparable datasets were not pooled with the primary mass-concentration dataset. Reporting of the predictive component was informed by relevant TRIPOD+AI principles [53], adapted to the literature-derived experimental toxicology; compliance with a clinical prediction-model framework was not claimed.

## 3. Results

### 3.1. Evidence Base and Final Analytical Datasets

**Figure 1:**
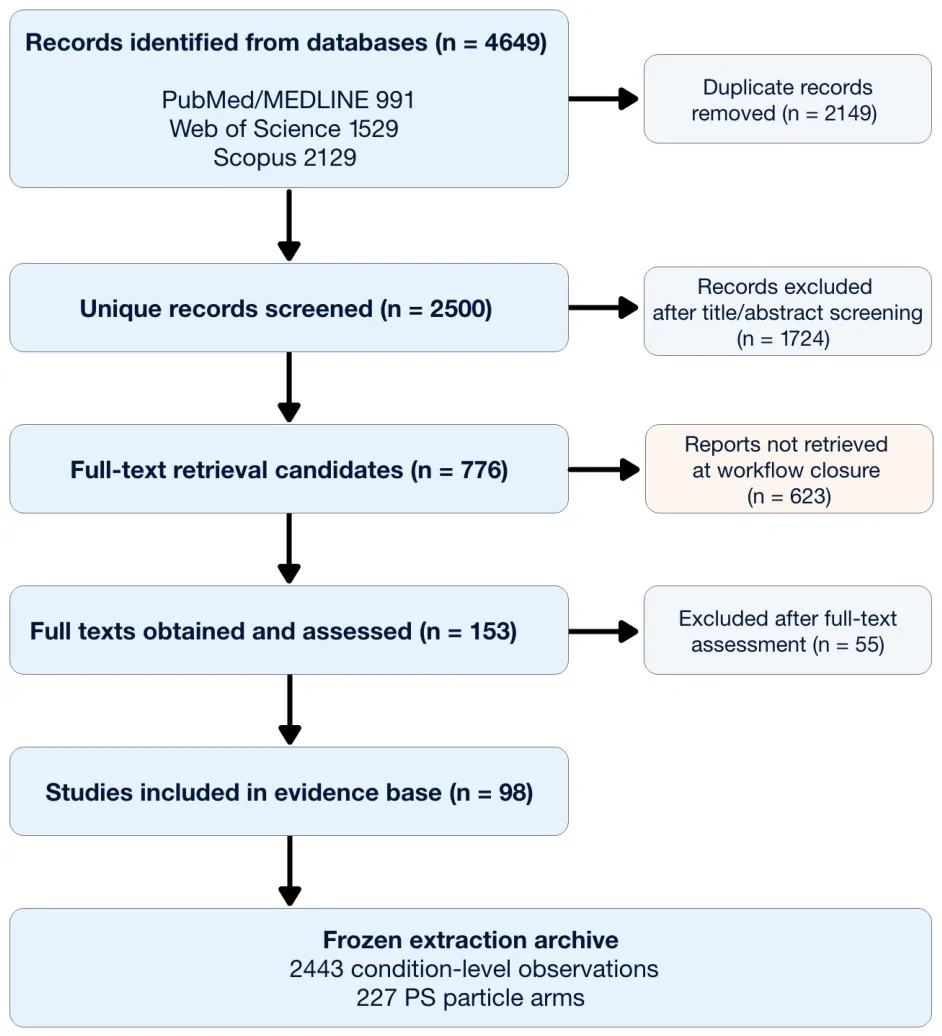
PRISMA-style flow diagram of database record identification, deduplication, title/abstract screening, full-text retrieval, eligibility assessment, and study inclusion. Reports not retrieved at workflow closure (*n* = 623) were recorded separately from full-text exclusions (*n* = 55); the archived records did not support a more detailed classification of retrieval failures. The 98 included studies contributed 227 polystyrene particle arms and 2443 condition-level observations.

Database searches identified 4649 records. Following the removal of 2149 duplicates, 2500 records underwent title and abstract screening, of which 1724 were excluded at this stage. Among the 776 records advanced to full-text retrieval, 153 full texts were successfully obtained and assessed. Of these, 98 studies met the final eligibility criteria, whereas 55 were excluded following full-text review. The remaining 623 candidate records were not retrieved by the end of the study-selection process and were recorded separately as reports not retrieved, rather than as exclusions following full-text assessment. A more detailed breakdown of the reasons for non-retrieval was not available from the archived screening records. The complete screening-flow audit is provided in Supplementary Table S20. The final frozen extraction archive comprised 227 particle arms and 2443 condition-level observations (Table 1; Figure 1).

Clarification of study and model denominators: The frozen R5 evidence inventory comprises 98 included studies. Four studies (PSNP-1791, PSNP-1903, PSNP-1907, and PSNP-1908) contributed no quantitative condition-level observations to the frozen extracted archive. A further 12 included studies contributed archived observations but no rows satisfying the primary-model-training eligibility criteria. Thus, 16 included studies did not enter the primary-modelling dataset (98 − 16 = 82), and the more restrictive ≤100 nm criterion yielded 60 studies in the strict sensitivity subset. The original PRISMA adjudication is preserved: 98 included studies and 55 full-text exclusions.

**Figure 2:**
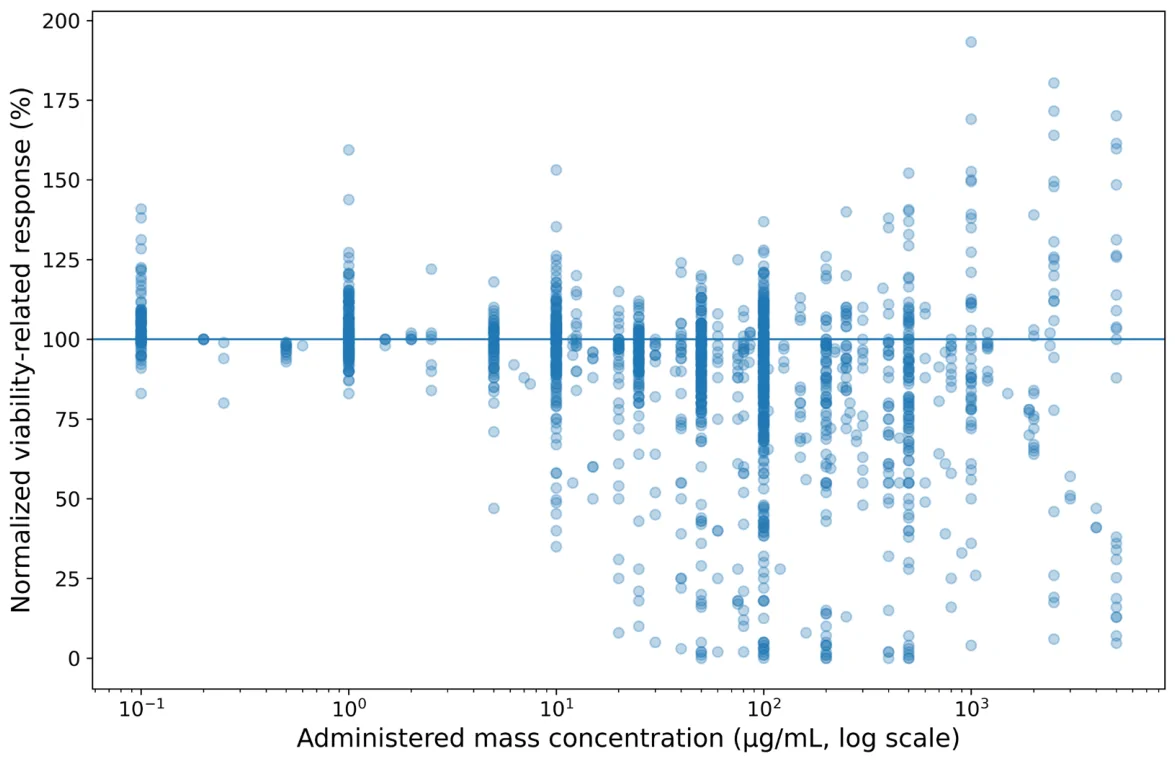
Primary analysis observations across administered mass concentration and normalized viability-related response. The horizontal reference line denotes 100% normalized response.

The Primary Mass-Concentration Dataset comprised 1701 non-zero exposure observations from 82 independent studies. Median administered mass concentration was 50 µg/mL (interquartile range (IQR), 10–125; range, 0.1–5000), median exposure duration was 24 h (IQR, 10–24; range, 0.333–672), and median normalized response was 96.0% (IQR, 82.0–102.0; range, 0–193.3). The relationship between administered mass concentration and normalized response is shown in Figure 2.

A numerical value for primary geometric diameter was available for 1629 observations, with a median of 100 nm (IQR, 50–200); 72 observations (4.2%) lacked a numerical primary geometric diameter. Human cell systems accounted for 1422 observations (83.6%). Figure-derived values represented 1641 observations (96.5%), whereas 60 observations from 19 studies (3.5%) were reported directly as numerical values.

The prespecified Strict ≤ 100 nm Dataset comprised 995 observations from 60 independent studies, of which 961 (96.6%) were figure-derived. Thirty-two training-eligible observations were reported exclusively as administered areal dose and were retained as a separate Secondary Areal-Dose Dataset. The Metric-Comparable Dataset comprised 141 observations from six independent studies, and all geometry-based conversions within this matched dataset met the predefined High dose-conversion-confidence criteria.

### 3.2. Benchmark Model Performance in the Primary Dataset

**Figure 3:**
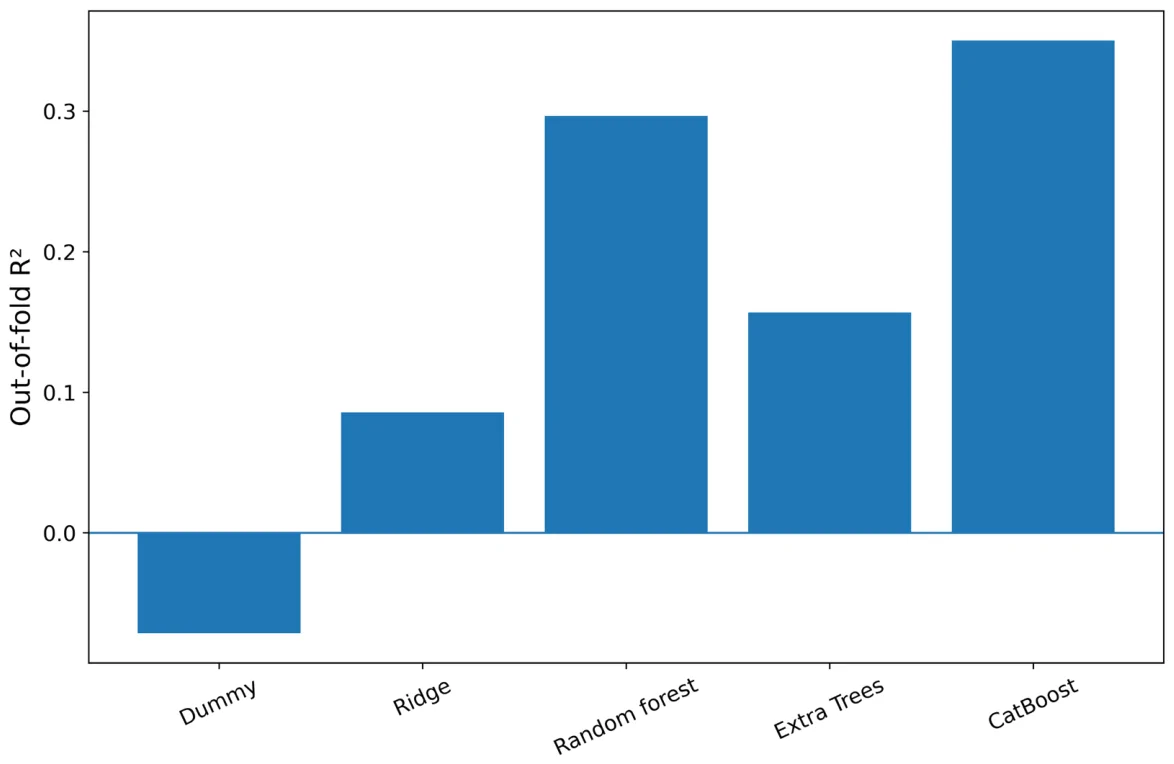
Out-of-fold R^2^ for benchmark models under study-grouped cross-validation. The horizontal reference line denotes R^2^ = 0.

Under study-grouped out-of-fold benchmark evaluation, the median dummy baseline yielded an MAE of 17.16 percentage points, an RMSE of 27.45, and an R^2^ of −0.071. Among the learned models, CatBoost achieved the lowest observation-level MAE (14.68), the lowest RMSE (21.38), and the highest observation-level R^2^ (0.350), whereas random forest achieved the lowest study-balanced MAE (15.42). Because the prespecified model-selection strategy prioritized study-balanced absolute error while also considering observation-level predictive performance, random forest was carried forward to the nested grouped analysis (Table 2; Figure 3). The benchmark results were used to select the model family for subsequent evaluation and were distinguished from the final performance estimates obtained through nested grouped cross-validation.

### 3.3. Nested Grouped Random Forest and Strict ≤ 100 nm Sensitivity Analysis

**Figure 4:**
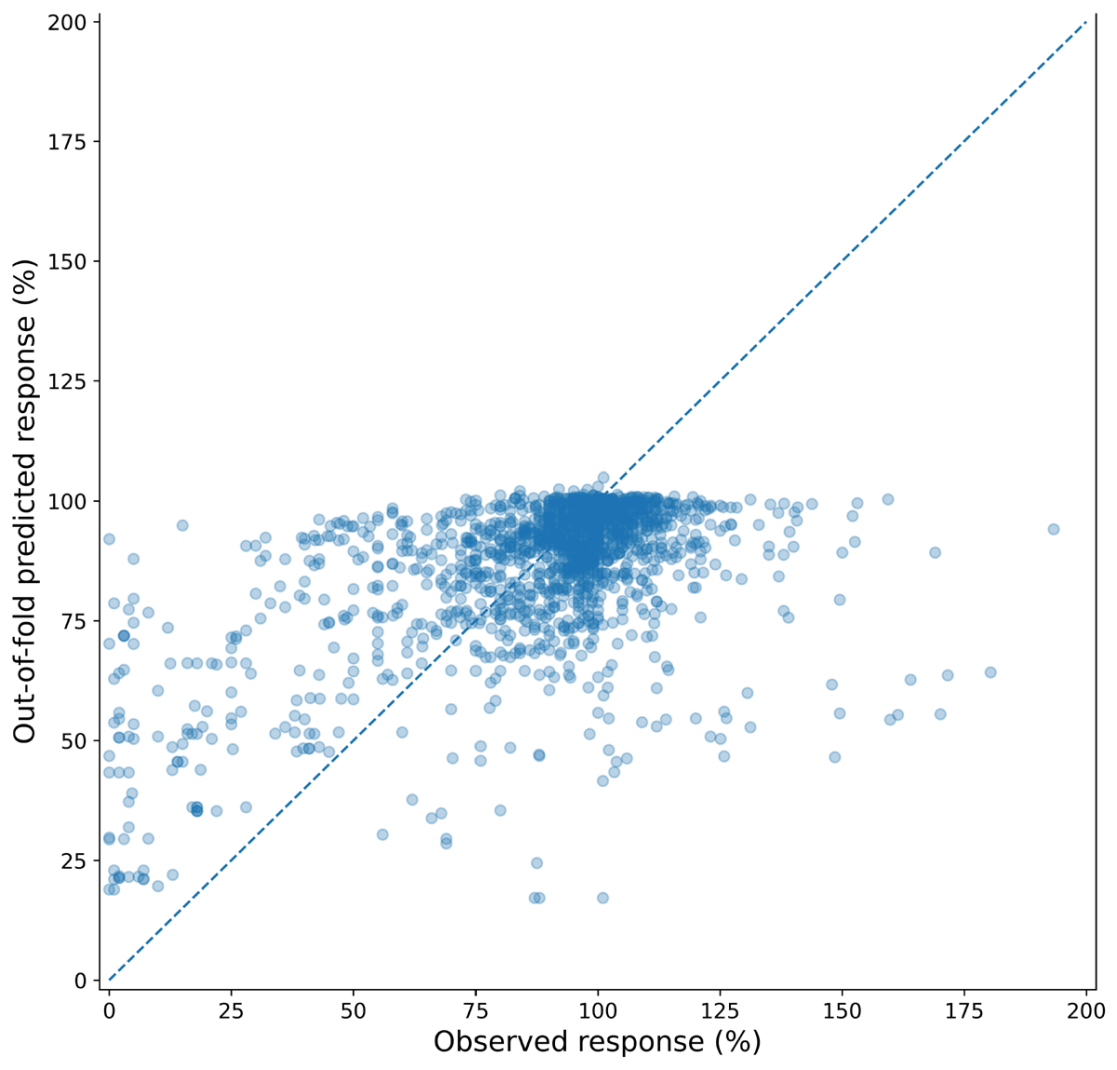
Observed versus out-of-fold predicted responses for the final nested grouped random forest in the primary dataset. The dashed diagonal line denotes perfect agreement (y = x).

In the full Primary Mass-Concentration Dataset, the final nested study-grouped random forest yielded an MAE of 15.36 percentage points (95% article-cluster-bootstrap confidence interval (CI), 12.78–19.41), an RMSE of 22.46 (17.94–28.42), and an R^2^ of 0.283 (0.033–0.461). The corresponding study-balanced MAE was 15.37, and the study-mean R^2^ was 0.095 (Table 3; Figure 4). The low study-mean R^2^ indicates that prediction remained limited when performance was summarized at the level of previously unseen source studies.

**Figure 5:**
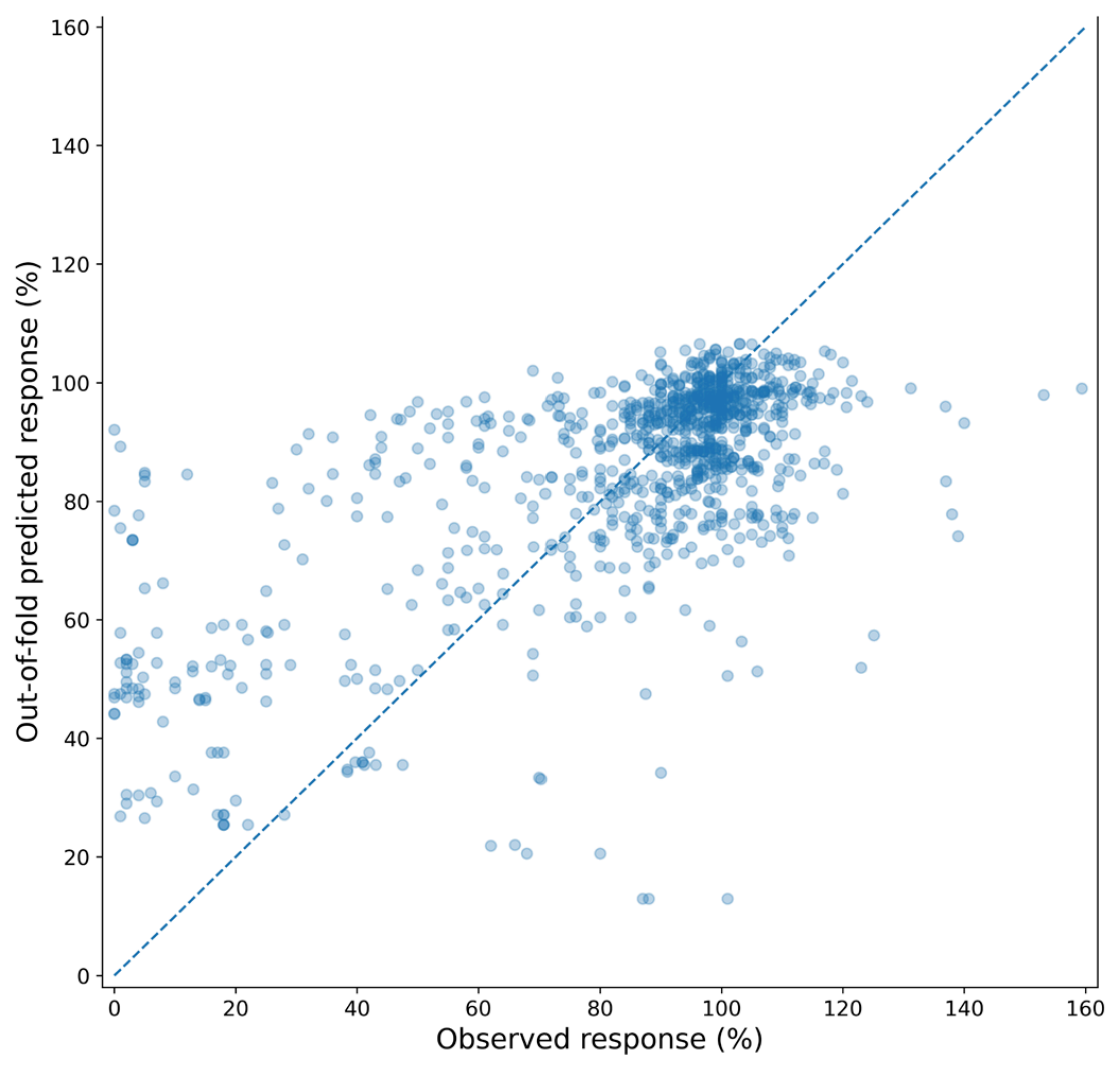
Observed versus out-of-fold predicted responses for the nested grouped random forest in the strict ≤ 100 nm subset. The dashed diagonal line denotes perfect agreement (y = x).

Within the prespecified ≤100 nm subset, the final nested study-grouped random forest yielded an MAE of 14.37 percentage points (95% CI, 12.30–17.07), an RMSE of 21.23 (18.48–24.67), and an R^2^ of 0.447 (0.281–0.556). The corresponding study-balanced MAE was 15.60, and the study-mean R^2^ was 0.322 (Table 3; Figure 5). Under the same nested study-grouped validation framework, restriction to ≤100 nm was associated with higher observation-level and study-mean R^2^ and slightly lower observation-level MAE and RMSE, but not with lower study-balanced MAE. Because the ≤100 nm restriction altered the composition of contributing studies, this comparison was interpreted as a prespecified sensitivity analysis rather than as a paired test of predictive superiority.

To examine whether this apparent improvement was attributable to nanoscale restriction itself or to changes in study composition, an additional post-review sensitivity analysis was conducted in the subset of 22 studies contributing observations both within and above the ≤100 nm threshold. Using identical held-out ≤100 nm observations from these shared studies, broad-domain and nanoscale-only training showed no clear predictive advantage for the nanoscale-restricted model. In the composition-controlled comparison, broad-domain and nanoscale-only training yielded observation-level MAEs of 19.83 and 19.77, R^2^ values of 0.292 and 0.274, and study-balanced MAEs of 19.70 and 20.61, respectively, on 447 identical held-out nanoscale observations (Supplementary Table S15). This result indicates that the more favourable observation-level performance seen in the full ≤100 nm subset should not be interpreted as independent evidence that restricting the training domain to ≤100 nm necessarily improves out-of-study prediction.

**Figure 6:**
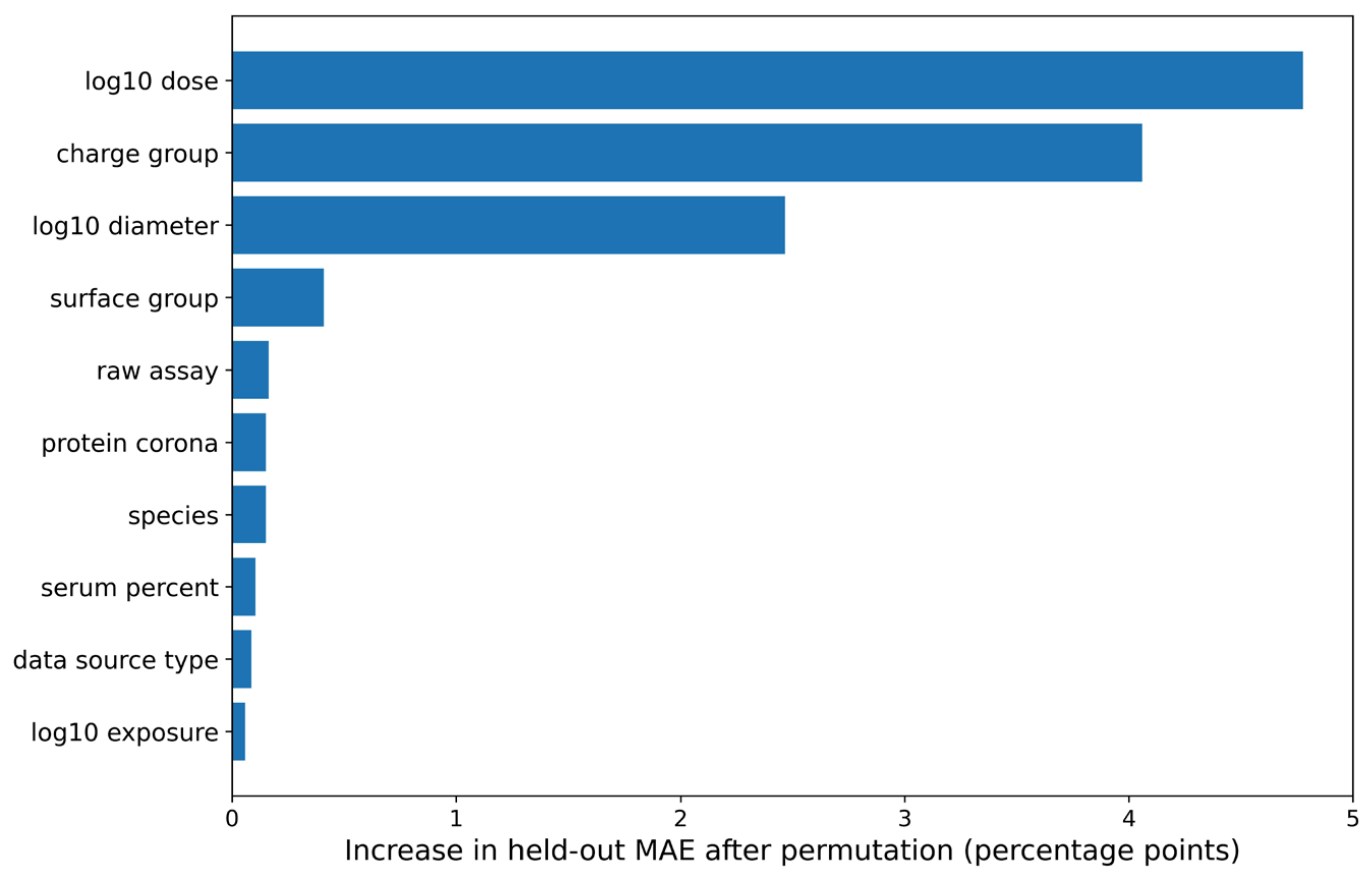
Top 10 held-out permutation importance values in the prespecified strict ≤ 100 nm model, expressed as the increase in MAE after feature permutation. Predictors are ordered by decreasing ΔMAE. Positive values indicate deterioration in held-out predictive accuracy after permutation.

Held-out permutation importance within the prespecified ≤100 nm model showed that permuting administered dose produced the largest increase in MAE (ΔMAE 4.78 percentage points), followed by the harmonized surface-charge group (ΔMAE 4.06) and primary particle diameter (ΔMAE 2.47). The surface-functionalization group showed a smaller contribution (ΔMAE 0.41). These values represent the mean deterioration in held-out predictive accuracy after permutation across the outer folds and should be interpreted as measures of predictive dependence rather than causal effects (Figure 6).

### 3.4. Matched Comparison of Mass, Particle-Number, and Geometric Surface-Area Dose Metrics

**Figure 7:**
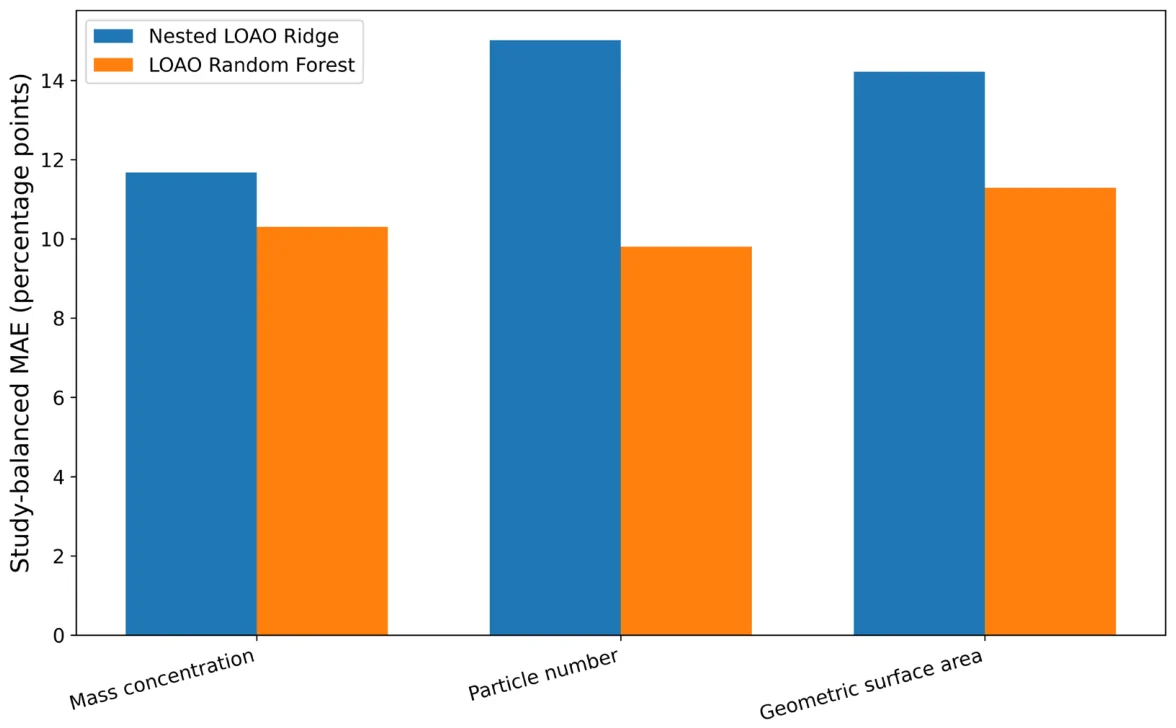
Study-balanced MAE for mass, particle-number, and geometric surface-area dose metrics in the matched geometry-comparable dataset.

In the matched geometry-comparable dataset, the relative performance of the three dose representations varied by model class. Under leave-one-article-out Ridge regression with grouped inner tuning, mass concentration yielded the lowest study-balanced MAE (11.68), compared with particle number (15.02) and geometric surface area (14.22); the corresponding observation-level MAEs were 7.52, 13.10, and 12.89, respectively. In the random-forest comparator, particle number yielded the lowest study-balanced MAE (9.80), compared with mass concentration (10.30) and geometric surface area (11.29) (Table 4; Figure 7).

For the random-forest comparison, the paired study-balanced contrast for particle number was ΔMAE (mass − number) = 0.50 percentage points (95% article-cluster-bootstrap CI, −1.87 to 2.87), whereas the corresponding contrast for geometric surface area was −0.99 percentage points (−3.00 to 0.50). The confidence interval for the apparent particle-number advantage therefore included zero, indicating substantial uncertainty around the direction of the difference. Because Ridge regression favoured mass concentration, whereas random forest showed only a small and uncertain advantage for particle number, no geometry-derived administered-dose representation demonstrated a consistent model-independent improvement over mass concentration.

In an additional post-review Ridge sensitivity analysis, the mass-only baseline yielded an observation-level MAE of 7.93 and a study-balanced MAE of 10.98. Adding primary particle diameter alone resulted in an observation-level MAE of 7.87 and a study-balanced MAE of 11.03, whereas including both diameter and the mass–diameter interaction yielded corresponding values of 7.34 and 10.43. The paired study-balanced MAE difference between the mass-only and interaction models was 0.55 percentage points (95% article-cluster-bootstrap CI, −0.73 to 2.13), providing no clear evidence of a consistent improvement across studies (Supplementary Table S17). Study-specific held-out errors varied: the interaction model reduced MAE in three of the six contributing studies and increased it in the remaining three (Supplementary Table S18). These additional results were obtained from a separate sensitivity analysis and were not substituted for the original Table 4 estimates.

### 3.5. Additional Robustness Analyses

The directly reported subset comprised 60 observations from 19 studies and yielded an MAE of 20.68, an R^2^ of −0.419, and a study-balanced MAE of 18.89. The assay-interference-control subset comprised 628 observations from 30 studies and yielded an MAE of 20.72, an R^2^ of 0.111, and a study-balanced MAE of 20.19. Both restrictions reduced the number of contributing studies and altered the composition of the analytical dataset; accordingly, these analyses were interpreted as domain-restriction diagnostics rather than as direct performance comparisons with the full Primary Mass-Concentration Dataset (Table 5). The negative R^2^ in the directly reported subset indicates limited predictive performance within that restricted dataset but does not, by itself, establish that figure-derived observations were inaccurate.

For a complementary paired diagnostic that did not require refitting, we stratified the frozen primary model’s out-of-fold predictions by Data_Source_Type and evaluated its 60 directly reported observations from 19 studies against dummy predictions derived exclusively from the training-fold directly reported responses. The primary-model OOF predictions yielded MAE 18.20 and R^2^ 0.135; on exactly the same held-out observations, a training-fold mean dummy yielded MAE 21.58 and R^2^ −0.212, and a training-fold median dummy yielded MAE 20.23 and R^2^ −0.200. These results are distinct from, and do not replace, the separately retrained direct-only restricted model (R^2^ −0.419); see Supplementary Table S21. The corresponding paired study-balanced MAE contrasts were +3.29 percentage points (mean dummy minus RF; 95% article-cluster-bootstrap CI, −1.73 to 8.33) and +1.16 percentage points (median dummy minus RF; 95% CI, −3.69 to 5.49). The paired study-balanced differences were not statistically distinguishable from zero given only 19 contributing studies.

Additional post-review analyses examined the influence of missing diameter values, predictor redundancy, and study quality. Excluding the 72 observations without a numerical primary particle diameter yielded a complete-case dataset of 1629 observations from 78 studies, with an MAE of 15.55, an R^2^ of 0.310, and a study-balanced MAE of 15.67. Removing raw assay and particle-size class from the predictor set retained 1701 observations from 82 studies and yielded an MAE of 15.58, an R^2^ of 0.236, and a study-balanced MAE of 15.77. Restriction to studies classified as High in both reporting and methodological quality retained 1575 observations from 70 studies and yielded an MAE of 15.42, an R^2^ of 0.270, and a study-balanced MAE of 15.55 (Supplementary Table S16). These analyses showed no consistent improvement across performance measures, although the complete-case and quality-restricted analyses involved different sets of observations and contributing studies.

An additional blinded human re-extraction assessment examined 30 selected figure-derived experimental conditions from ten source articles. Numerical values could be independently recovered for 23 conditions from eight articles, whereas seven conditions could not be quantified reliably. Among the 23 readable comparisons, the mean absolute difference was 1.43 percentage points (median, 0.90; RMSE, 2.45, on the raw figure-response scale), and 20 of 23 values (87.0%) differed by no more than two percentage points. These findings describe agreement within the selected readable conditions rather than extraction accuracy across the full dataset. One additional targeted quality-control observation was assessed separately and excluded from the 30-condition comparison. Remaining discrepancies in source-figure interpretation and response normalization were retained for further adjudication without modifying the frozen analytical dataset (Supplementary Table S19).

## 4. Discussion

This study yielded two complementary findings that are directly relevant to predictive toxicology of PS micro- and nanoplastics. First, restriction to the prespecified ≤100 nm domain was associated with higher observation-level and study-mean R^2^ and slightly lower observation-level prediction error, although study-balanced MAE did not improve. Second, replacing nominal mass concentration with geometry-derived particle-number or geometric surface-area concentration did not provide a consistent predictive advantage across model classes in the matched geometry-comparable dataset. Together, these findings suggest that the apparent predictive gains associated with nanoscale restriction require consideration of study composition, whereas changing administered-dose representation alone does not provide a model-independent advantage.

The higher out-of-study R^2^ and slightly lower observation-level error observed in the ≤100 nm analysis should not be interpreted as evidence that smaller PS particles are intrinsically more toxic. The nanoscale subset differed from the broader dataset in both particle-size range and contributing study composition; accordingly, this analysis represents a prespecified sensitivity assessment rather than a paired test of predictive superiority. In an additional post-review analysis restricted to 22 studies contributing observations on both sides of the ≤100 nm threshold, broad-domain and nanoscale-only models were evaluated on the same 447 held-out nanoscale observations. Nanoscale-only training did not improve study-balanced MAE (20.61 versus 19.70) or R^2^ (0.274 versus 0.292) relative to broad-domain training, and the corresponding paired uncertainty intervals included zero (Supplementary Table S15). Particle-size-dependent heterogeneity remains biologically plausible because particle size influences cellular uptake pathways, membrane interactions, protein adsorption, intracellular trafficking, diffusion and sedimentation behaviour, and the particle number and total surface area represented by a fixed mass dose [4,12,20,21,22,23,24,25,26,40,41]. Extending the modelling domain toward 1 µm encompasses particles with increasingly different transport and interaction regimes and may therefore introduce structural heterogeneity that cannot be fully accommodated by a single cross-study model. However, the composition-controlled results do not establish that restricting the training domain to ≤100 nm improves prediction independently of the studies represented.

The geometry-derived administered-dose analysis addressed a related but analytically distinct particle-characterization and dosimetry question: whether a dose representation that preserves particle geometry could recover predictive information lost when PS particles of different sizes are expressed solely by mass concentration. Particle-number and geometric surface-area metrics are mechanistically attractive because they retain geometric information that mass alone does not capture [13,14,15,16,17,18,19]. However, only six independent studies provided sufficient characterization to compare all three dose representations on identical experimental observations. Within this constrained matched dataset, Ridge regression favoured mass concentration, whereas the random-forest comparator showed only a small particle-number advantage, and the corresponding paired article-cluster-bootstrap interval crossed zero. The relative ordering of dose metrics was therefore model dependent rather than reproducible across model classes. A post-review Ridge sensitivity analysis further showed that adding primary diameter and its interaction with mass concentration reduced study-balanced MAE from 10.98 to 10.43. However, the paired improvement of 0.55 percentage points had a 95% article-cluster-bootstrap CI of −0.73 to 2.13, and the interaction model improved prediction in only three of the six contributing studies (Supplementary Tables S17 and S18). These supplementary estimates arose from a separate model specification and do not replace the original matched-comparison results. Moreover, geometry-derived administered-dose calculations depend on reported primary diameter, morphology, and material density while remaining distinct from delivered cellular dose, which additionally depends on agglomeration, effective density, medium properties, liquid-column height, diffusion, sedimentation, and exposure geometry [24,25,26,27,28,29].

These findings also identify a particle-characterization bottleneck at the interface of polymer science and micro-/nanoplastic toxicology: improving dose representation requires not only alternative metrics but also sufficiently complete reporting of polymer-particle properties and exposure conditions. A recent systematic assessment of exposure metrics in the MP/NP literature identified substantial heterogeneity in reported dose descriptors and showed that missing experimental information frequently prevented reconstruction of comparable administered doses [54]. Although that analysis focused on murine studies, the methodological problem is directly relevant to the literature-derived in vitro modelling. Geometry-derived metrics cannot be calculated reliably or compared consistently when the particle characterization required to derive them is unavailable or incompletely reported. The present findings therefore do not establish that geometry-derived metrics should replace mass concentration as the practical cross-study reference under the present, limited evidence base. Rather, mass concentration should be interpreted alongside particle size, morphology, density, and surface characteristics, while geometry-derived metrics remain valuable secondary representations when the required physicochemical information is available.

The study-grouped validation design was central to the interpretation of predictive performance. The literature-derived toxicology datasets frequently contain multiple dose levels, exposure durations, particle formulations, and cellular conditions originating from the same publication. Random row-wise splitting can therefore place closely related observations in both training and test sets, producing performance estimates that partly reflect within-study similarity rather than transportability to an independent study [37,38,39]. By grouping every resampling stage by Article_ID and applying inverse-study-frequency fitting weights, the present framework instead evaluated whether exposure–response relationships learned from one set of publications generalized to previously unseen studies. The difference between the number of row-level observations and the number of independent contributing studies illustrates why study-level separation is particularly important in the literature-derived predictive toxicology. Despite an observation-level R^2^ of 0.283 in the primary model, the study-mean R^2^ was only 0.095, indicating limited ability to explain differences in mean responses across held-out studies. The models should therefore not be regarded as providing reliable quantitative predictions for an arbitrary new experimental setting.

Within the nanoscale-restricted model, permutation of administered dose produced the largest deterioration in held-out predictive accuracy, followed by the harmonized surface-charge group and primary particle diameter. These polymer-particle descriptors are biologically plausible contributors to model performance because particle size and surface charge are established determinants of PS particle uptake, membrane association, intracellular localization, and cellular response [4,7,12,20,21,22,23]. Nevertheless, permutation importance quantifies predictive dependence rather than biological causality. A predictor may appear important because it captures correlated differences in particle source, cellular system, assay architecture, or study design. Accordingly, the present analysis identifies material and exposure descriptors that contributed to held-out prediction but cannot establish that these variables independently caused the observed cytotoxic responses.

The robustness analyses further emphasize the importance of study composition. Directly reported numerical outcomes represented only a small fraction of the evidence base, and restriction to these observations yielded poorer grouped predictive performance (R^2^ = −0.419; 60 observations from 19 studies). Similarly, restricting the dataset to observations from studies reporting assay-interference controls did not produce stronger out-of-study prediction. These findings should not be interpreted as evidence that directly reported or better-controlled experiments are less informative or of lower methodological quality. Both restrictions substantially reduced the number of contributing studies and changed the analytical domain; they were therefore treated as domain-restriction diagnostics rather than as direct quality comparisons. Additional post-review analyses provided similar grounds for caution. Excluding 72 observations with missing primary diameter yielded an MAE of 15.55, compared with 15.36 in the primary model, although the contributing studies differed. Removing potentially redundant assay and particle-size predictors yielded an MAE of 15.58 and an R^2^ of 0.236, while restriction to studies classified as High in both reporting and methodological quality yielded an MAE of 15.42 and an R^2^ of 0.270 (Supplementary Table S16). These analyses did not identify a consistent improvement across performance measures and do not eliminate possible effects of missingness, predictor dependence, or study quality.

More broadly, the study highlights a reporting challenge at the interface of polymer characterization and toxicology. Figure-derived observations accounted for 96.5% of the primary-modelling dataset, indicating that condition-level numerical data are rarely available in directly reusable form. Although figure digitization enabled recovery of a substantially larger evidence base, it necessarily introduces additional measurement uncertainty and limits recovery of variance information. An additional blinded human re-extraction assessment examined 30 selected figure-derived conditions, of which 23 yielded independently readable numerical values and seven could not be quantified reliably. Among the 23 readable comparisons, the mean absolute difference was 1.43 percentage points, with 20 values differing by no more than two percentage points. This limited assessment cannot establish extraction accuracy across the complete figure-derived dataset, particularly where condition assignment or normalization remains uncertain. The broader literature similarly identifies incomplete and heterogeneous exposure reporting as a major barrier to quantitative synthesis and risk-relevant interpretation [54]. Future experimental studies of polymer particles would increase their secondary-use value by reporting condition-level numerical outcomes, primary geometric diameter with measurement uncertainty, morphology, material density and its source, surface functionalization and charge, medium volume and growth surface area, serum conditions, replicate structure, assay-interference controls, untreated-control values, and sufficient information to distinguish administered from potentially delivered dose.

Several additional limitations should be considered. First, 623 records in the full-text candidate pool could not be retrieved before the study-selection workflow closed; the final evidence base should therefore not be regarded as exhaustive. The archived screening records did not permit a reliable breakdown of the specific reasons for non-retrieval. Second, the high proportion of figure-derived observations introduces extraction uncertainty despite explicit source coding and prespecified digitization quality control. Third, the geometry-comparable analysis contained 141 matched observations but only six independent studies, meaning that the amount of row-level information substantially exceeded the number of independent units supporting between-metric inference. Fourth, the present models represented administered rather than delivered cellular dose, limiting direct translation from controlled laboratory exposure to human or environmentally realistic exposure scenarios. Recent analyses of the microplastic toxicology literature have highlighted substantial gaps between experimental exposure designs and realistic exposure conditions, including differences in dose magnitude, exposure duration, particle composition, and environmental complexity [55]. Fifth, residual heterogeneity remained across cell systems, assay platforms, serum conditions, exposure durations, and particle preparations. Finally, the present study provides study-grouped internal validation across independent publications rather than validation in a prospectively designed external experimental cohort.

These limitations are balanced by several methodological strengths. The analysis was based on a frozen condition-level extraction archive, maintained a strict distinction among mass concentration, administered areal dose, and geometry-derived administered-dose metrics, preserved source-reported responses above 100%, and avoided unsupported pseudo-controls, intermediate doses, and unit conversions. Model development and performance estimation were separated through nested study-grouped validation, while inverse-study-frequency weighting and study-balanced performance measures reduced the influence of articles contributing disproportionately large numbers of observations. The nanoscale analysis was prespecified, and dose-metric comparisons were conducted on identical experimental observations rather than independently filtered datasets. Additional post-review analyses examined study-composition effects, alternative Ridge specifications, missing diameter information, predictor redundancy, and study quality without replacing the original primary analyses. These design choices deliberately favoured conservative estimates of transportability over maximization of apparent predictive performance. The resulting framework therefore provides a reproducible data-driven approach for examining polymer-particle property–exposure–response relationships while explicitly respecting study clustering, reporting heterogeneity, and applicability-domain constraints. Prospective multi-laboratory experiments using standardized particle panels, measured in-medium particle behaviour, harmonized viability assays, and openly available condition-level data are needed to establish whether the observed relationships generalize to new experimental systems and whether alternative administered-dose metrics provide reproducible predictive benefits.

## 5. Conclusions

In this literature-derived analysis of mammalian in vitro PS cytotoxicity data, the prespecified nanoscale-restricted subset (≤100 nm) showed higher observation-level and study-mean R^2^ than the broader 1–1000 nm analysis, together with slightly lower observation-level MAE and RMSE. The full-domain model yielded an R^2^ of 0.283 and a study-mean R^2^ of 0.095, whereas the nanoscale-restricted model yielded an R^2^ of 0.447 and a study-mean R^2^ of 0.322 under nested study-grouped validation. However, study-balanced MAE was 15.37 in the primary dataset and 15.60 in the strict subset, indicating that restriction to the narrower particle-size domain did not improve all measures of predictive error. The low study-mean R^2^ in the full-domain model also indicates limited predictive performance at the level of independent studies. Furthermore, a post-review composition-controlled analysis involving 22 studies contributing both particle-size ranges showed no clear advantage for nanoscale-only training when evaluated on identical held-out ≤100 nm observations. The apparent improvement in the prespecified nanoscale subset therefore cannot be attributed confidently to particle-size restriction independently of study composition.

Geometry-derived administered-dose metrics retained physicochemical information not represented by mass concentration alone, but the present six-study matched evidence base did not demonstrate a consistent predictive advantage of particle-number or geometric surface-area concentration over mass concentration. For cross-study modelling of PS micro- and nanoplastic cytotoxicity, mass concentration therefore remains a practical reference metric within the current evidence base, provided that it is interpreted alongside particle size and surface characteristics and that model validation preserves study-level independence. This practical role should not be interpreted as evidence that mass concentration is intrinsically the most biologically relevant dose metric. Moreover, geometry-derived metrics describe administered dose rather than the dose delivered to cells. More complete and standardized reporting of condition-level viability outcomes, primary particle size and morphology, material density, surface functionalization and charge, and exposure descriptors will be important for determining whether geometry-derived administered-dose metrics can provide reproducible cross-study predictive gains. More broadly, the results support treating particle-size domain definition and polymer-particle characterization as central components of data-driven analysis rather than as secondary reporting details. Prospective cross-laboratory validation with standardized particle characterization and exposure conditions will be necessary to establish the generalizability of these findings.

## Figures and Tables

**Table 1:** Final evidence base and primary analytical datasets.

Characteristic	Value	Characteristic	Value
Full-text candidate pool	776	Full texts assessed	153
Final included studies	98	Full-text exclusions	55
Not retrieved/unresolved	623	Frozen observations	2443
Particle arms	227	Primary-model observations	1701
Primary-model studies	82	Strict ≤ 100 nm observations	995
Strict ≤ 100 nm studies	60	Geometry-comparable observations	141
Geometry-comparable studies	6	Areal-dose observations	32
Dose, median (IQR)	50 (10–125) µg/mL	Exposure, median (IQR)	24 (10–24) h
Response, median (IQR)	96.0 (82.0–102.0)%	Diameter, median (IQR)	100 (50–200) nm

Note: IQR, interquartile range.

**Table 2:** Benchmark study-grouped model performance in the primary mass-concentration dataset.

Model	MAE	RMSE	R^2^	Study-Balanced MAE
Median dummy	17.16	27.45	−0.071	17.09
Ridge	19.19	25.36	0.086	18.29
Random forest	15.18	22.24	0.297	15.42
Extra Trees	16.98	24.36	0.157	16.32
CatBoost	14.68	21.38	0.350	15.65

Note: MAE, mean absolute error; RMSE, root mean square error; R^2^, coefficient of determination.

**Table 3:** Final nested grouped random-forest performance.

Dataset	Rows/Studies	MAE (95% CI)	RMSE (95% CI)	R^2^ (95% CI)	Study Balanced MAE	Study Mean R^2^
Primary ≤1000 nm	1701/82	15.36 (12.78–19.41)	22.46 (17.94–28.42)	0.283 (0.033–0.461)	15.37	0.095
Strict ≤100 nm	995/60	14.37 (12.30–17.07)	21.23 (18.48–24.67)	0.447 (0.281–0.556)	15.60	0.322

Note: CI, confidence interval; MAE, mean absolute error; RMSE, root mean square error; R^2^, coefficient of determination.

**Table 4:** Matched geometry-comparable dose-metric analysis (141 observations from six studies).

Model	Dose Metric	MAE	Study-Balanced MAE	R^2^
Nested LOAO Ridge	Mass concentration	7.52	11.68	0.186
Nested LOAO Ridge	Particle number	13.10	15.02	−0.575
Nested LOAO Ridge	Geometric surface area	12.89	14.22	−0.404
LOAO RF	Mass concentration	8.37	10.30	0.091
LOAO RF	Particle number	7.82	9.80	0.129
LOAO RF	Geometric surface area	8.49	11.29	0.052

Note: LOAO, leave-one-article-out; RF, random forest; MAE, mean absolute error; R^2^, coefficient of determination.

**Table 5:** Exploratory robustness subsets.

Subset	Rows/Studies	MAE	Study-Balanced MAE	R^2^	Study-Mean R^2^
Directly reported only	60/19	20.68	18.89	−0.419	−0.455
Assay-interference control reported	628/30	20.72	20.19	0.111	−0.736

Note: MAE, mean absolute error; R^2^, coefficient of determination.

## Data Availability

The original contributions presented in this study are included in the article/Supplementary Material. Further inquiries can be directed to the corresponding author.

## Supplementary Materials

The following supporting information can be downloaded at: https://www.mdpi.com/article/10.3390/polym18202575/s1. Table S1. Evidence-identification and full-text workflow summary; Table S2. Operational eligibility framework; Table S3. Final analytical datasets; Table S4. Deterministic derived-variable mappings used in the analysis-ready dataset; Table S5. Full study-grouped benchmark performance in the primary mass-concentration dataset; Table S6. Final nested grouped random-forest performance and article-cluster-bootstrap 95% confidence intervals; Table S7. Outer-fold performance and selected random-forest hyperparameters; Table S8. Full held-out permutation importance in the strict ≤ 100 nm nested model; Table S9. Matched geometry-comparable dose-metric performance (141 observations, six studies); Table S10. Paired study-balanced MAE contrasts in the matched geometry-comparable random-forest analysis; Table S11. Exploratory robustness subsets; Table S12. R5 post-freeze correction provenance; Table S13. Characteristics of the final included studies; Table S14. Reproducibility files used to generate the final manuscript results; Table S15. Common-study nanoscale sensitivity on identical held-out ≤100 nm observations; Table S16. Primary and post-review robust-configuration performance; Table S17. Reviewer-requested post hoc Ridge interaction sensitivity; Table S18. Article-specific errors in the post hoc matched Ridge sensitivity; Table S19. Blinded independent human figure re-extraction: agreement and outstanding QC; Table S20. Full evidence-identification and screening-flow audit; Table S21. Frozen R5 primary RF OOF predictions versus training-fold dummy predictors on the identical directly reported subset (60 observations, 19 studies); Table S22. Study-level MAE of the frozen R5 full-primary nested RF OOF predictions, restricted to the six geometry-comparable articles (141 observations).

## Abbreviations

The following abbreviations are used in this manuscript:

PS	Polystyrene
MAE	Mean absolute error
RMSE	Root mean square error
CI	Confidence interval
IQR	Interquartile range
LOAO	Leave-one-article-out
RF	Random forest
MTT	3-(4,5-dimethylthiazol-2-yl)-2,5-diphenyltetrazolium bromide
MTS	3-(4,5-dimethylthiazol-2-yl)-5-(3-carboxymethoxyphenyl)-2-(4-sulfophenyl)-2H-tetrazolium
XTT	2,3-bis(2-methoxy-4-nitro-5-sulfophenyl)-2H-tetrazolium-5-carboxanilide
WST	Water-soluble tetrazolium
CCK-8	Cell Counting Kit-8
DOI	Digital object identifier
PMID	PubMed identifier
PRISMA	Preferred Reporting Items for Systematic Reviews and Meta-Analyses
PRISMA-S	PRISMA extension for reporting literature searches
QSAR	Quantitative structure–activity relationship
LDH	Lactate dehydrogenase
TRIPOD+AI	Transparent Reporting of a multivariable prediction model for Individual Prognosis or Diagnosis, extended for artificial intelligence
