# Supplementary material for: Study-Grouped Predictive Toxicology of In Vitro Polystyrene Micro- and Nanoplastic Cytotoxicity: Geometry-Derived Administered-Dose Metrics and a Nanoscale-Restricted Sensitivity Analysis
Source: Polymers (Basel). 2026 Oct 19;18(20):2575. doi: 10.3390/polym18202575 (PMC13709059; doi:10.3390/polym18202575)
Supplement: Supplementary file 1 [file polymers-18-02575-s001.zip › polymers-4609234-supplementary.pdf]

Supplementary Material

Study-Grouped Predictive Toxicology of In Vitro Polystyrene Micro- and Nanoplastic Cytotoxicity: Geometry-Derived Administered-Dose Metrics and a Nanoscale-Restricted Sensitivity Analysis

This Supplementary Material is aligned with the final R5-corrected analysis used in the manuscript. All counts, model-performance values, study-grouped resampling specifications, dose-metric comparisons, and sensitivity analyses correspond to the final analytical datasets: 98 eligible studies, 227 particle arms, 2,443 condition-level observations, 1,701 primary mass-concentration observations from 82 studies, and 995 strict ≤100 nm observations from 60 studies.

Contents

Supplementary Methods S1. Evidence identification and search audit
Supplementary Methods S2. Eligibility framework and screening rules
Supplementary Methods S3. Data extraction, harmonization, and figure-derived values
Supplementary Methods S4. Geometry-derived administered-dose representation and geometry-comparable subset
Supplementary Methods S5. Analysis-dataset construction
Supplementary Methods S6. Predictive modelling and resampling specifications
Supplementary Methods S7. Performance metrics and uncertainty
Supplementary Methods S8. Reproducibility and R5 correction provenance
Supplementary Methods S9. Post-review sensitivity analyses and source-figure audit
Supplementary Tables S1–S12
Supplementary S1. Full search syntax
Supplementary S2. Included-study summary (Table S13)
Supplementary data and reproducibility manifest (Table S14)
Post-review sensitivity analyses and audit results (Tables S15–S22)

Supplementary Methods S1. Evidence identification and search audit

The evidence-identification workflow targeted mammalian in vitro studies reporting quantitative viability, survival-, or metabolic-activity responses following exposure to polystyrene (PS) particles. PubMed/MEDLINE, Web of Science Core Collection, and Scopus were searched from database inception through 13 September 2026. The search architecture was deliberately sensitive and combined three complementary branches: (i) a modern PS-nanoplastic branch, (ii) a legacy PS latex/bead/microsphere branch, and (iii) an abbreviation safety-net branch including PSNP, PS-NP, and PS-NPL variants. Terms related to machine learning, particle number, or surface area were not required for retrieval so that study identification was independent of the later analytical framework.

Search records were merged and deduplicated hierarchically using exact DOI, exact PMID, and normalized-title matching when identifiers were compatible and publication years differed by no more than one year. Records with conflicting identifiers or ambiguous near-matches were retained for manual review rather than merged automatically. Across the three databases, 4,649 records were identified (PubMed/MEDLINE, n = 991; Web of Science, n = 1,529; Scopus, n = 2,129). After removal of 2,149 duplicate records, the final title/abstract screening master contained 2,500 unique records; 1,724 records were excluded following title/abstract screening, and 776 records advanced to full-text retrieval. At workflow closure, 153 full texts had been obtained and assessed, 98 studies were included, 55 were excluded after full-text review, and 623 candidate records remained not retrieved or unresolved. These 623 records were distinguished from studies excluded after full-text assessment because their eligibility

could not be determined without the corresponding full texts. The archived screening records did not support a reliable breakdown of specific reasons for non-retrieval. The complete identification and screening flow is documented in Supplementary Table S20 and Figure 1 of the main manuscript.

A Web of Science search-history audit documented 1,310 records from the modern branch, 334 from the legacy latex/bead/microsphere branch, and 267 from the abbreviation branch. The union of the first two branches contained 1,503 records, while the abbreviation branch added 26 records not retrieved by those branches, giving 1,529 records for the three-branch Web of Science union. These branch yields are reported as a search-audit check and should not be summed across databases because records overlap substantially across sources.

Supplementary Methods S2. Eligibility framework and screening rules

Eligibility was operationally based on PS composition, primary particle diameter, mammalian in vitro biological systems, and availability of a compatible quantitative viability-related response. The broad evidence-capture domain was 1–1000 nm, while a prespecified ≤100 nm subset was retained for nanoscale-restricted sensitivity analysis.

Frozen-screening clarification: the 98 included studies are the R5 evidence-inventory denominator; PSNP-1791, PSNP-1903, PSNP-1907, and PSNP-1908 contributed no eligible quantitative model rows in the frozen R5 extraction. Accordingly, these four reports do not enter the study counts for the primary, strict, or matched geometry models. We did not change the original full-text exclusion or inclusion classification (55 and 98, respectively).

Commercially engineered particles, laboratory-generated/degraded PS, and environmentally derived/weathered PS were eligible when a separable PS-specific arm satisfied the remaining criteria. Title and abstract screening were performed independently by two reviewers using Include, Exclude, and Unclear categories. Exclusion required clear evidence of ineligibility. Missing particle size, incomplete physicochemical characterization, absence of an explicitly stated viability endpoint in the abstract, or uncertainty regarding an eligible mammalian in vitro arm was not sufficient for exclusion; these records were classified as Unclear and advanced to full-text retrieval rather than being assigned a final eligibility decision at the screening stage. Reviewer calibration used an initial 100-record pilot and, where needed, an independent 50-record confirmation set. The operational progression threshold was ≥85% raw agreement and Cohen's κ ≥0.60 for Advance versus Exclude, with both Include and Unclear classified as Advance. Records classified as Include or Unclear proceeded to the full-text retrieval stage, whereas records classified as Exclude did not. Disagreements affecting progression were resolved by consensus or, where necessary, adjudication. Full-text eligibility assessment was restricted to reports successfully

obtained; records not retrieved at workflow closure were not assigned full-text eligibility exclusion reasons.

At full-text assessment, eligibility required a mammalian in vitro exposure to PS particles within the defined size range, at least one non-zero exposure condition, and an extractable quantitative viability-, survival-, or metabolic-activity outcome suitable for normalization to the corresponding untreated control. Studies reporting membrane-damage endpoints such as lactate dehydrogenase (LDH) release alone were not considered eligible. Mixed exposures were retained only when a PS-alone condition could be evaluated separately. When numerical outcomes were available only in graphical form, studies remained eligible if the relevant experimental conditions could be identified and the response values reliably extracted. Non-English articles were retained, with machine-assisted translation used where necessary and critical numerical information independently checked.

Supplementary Methods S3. Data extraction, harmonization, and figure-derived values

Extraction was performed at the condition level: one row represented a distinct article, particle arm, cell model, assay, exposure duration, administered dose, and response combination. Untreated controls were used for normalization and quality control but were not entered as dose = 0 training observations. Values above 100% of control were retained as reported. Primary geometric diameter was kept distinct from hydrodynamic diameter, and hydrodynamic diameter was not substituted when primary diameter was missing.

Figure-derived values were extracted only when the plotted condition, dose, exposure duration, and experimental group could be mapped unambiguously to the source text and figure caption. Numerical precision was limited to the graphical resolution supported by the source. Unsupported pseudo-controls, visually indeterminate intermediate doses, and unsupported unit conversions were not created. The final primary dataset contained 1,641 figure-derived observations (96.5%) and 60 directly reported observations (3.5%; 19 studies). Seven previously unresolved studies were recovered from supplementary figures and contributed 219 figure-derived observations to the complete frozen extraction archive.

An additional post-review quality-control assessment involved independent re-extraction of 30 selected figure-derived experimental conditions from ten source articles (three conditions per article). A second human researcher, blinded to the originally archived response values, examined the corresponding source figures and experimental conditions. Numerical estimates were recorded only when the relevant figure panel, axis, and experimental condition could be identified with sufficient confidence. Conditions that could not be quantified reliably were retained as unresolved rather than assigned imputed values. Of the 30 attempted readings, 23 yielded independently readable numerical values from eight articles, whereas seven remained non-quantifiable. Agreement was evaluated on the raw figure-response scale,

separately from model prediction performance. The independent extraction procedure, agreement statistics, and outstanding source-level quality-control issues are detailed in Supplementary Methods S9.6 and Supplementary Table S19.

Supplementary Methods S4. Geometry-derived administered-dose representation and geometry-comparable subset

Geometry-derived administered-dose calculations were restricted to observations with sufficient primary-particle geometry and density provenance. For a spherical particle of primary diameter d and material density ρ, particle volume was $V = \pi d^3/6$ and single-particle mass was $m = \rho\pi d^3/6$. For administered mass concentration C_m, particle-number concentration was $N = C_m/m = 6C_m/(\rho\pi d^3)$, and geometric surface-area concentration was $A = N\pi d^2 = 6C_m/(\rho d)$. Calculations used internally consistent units (d in cm; ρ in g/cm³; C_m in g/mL), yielding N in particles/mL and A in cm²/mL. These geometry-derived metrics describe nominally administered particle-number and surface-area concentrations under idealized spherical-particle assumptions. They do not account for agglomeration, diffusion, sedimentation, or deposition at the cellular interface and therefore should not be interpreted as measures of delivered cellular dose.

Conversions were not enabled solely by assuming a generic PS density. Density had to be source-supported within the extraction/QC record, and DLS-only size estimates were not accepted as primary geometric diameter for conversion. The final geometry-comparable dataset comprised 141 identical experimental observations from six studies for which mass concentration, particle-number concentration, and geometric surface-area concentration could all be represented. Because all three metrics were evaluated on the same observations, differences in model performance were not attributable to changes in sample composition.

Supplementary Methods S5. Analysis-dataset construction

The Primary Mass-Concentration Dataset included non-zero mass-concentration observations with exposure duration >0, a quantitative normalized response, and model-training eligibility. The strict sensitivity dataset applied the locked ≤100 nm flag to the primary dataset. Areal-dose observations reported in µg/cm² were retained separately and were never pooled with mass concentration. Geometry-comparable observations were likewise evaluated as a separate matched analysis. The resulting Primary Mass-Concentration Dataset comprised 1,701 observations from 82 studies, while the Strict ≤100 nm Dataset comprised 995 observations from 60 studies. The Secondary Areal-Dose Dataset contained 32 observations from a single study and was not used for independent study-grouped predictive modelling; the Metric-Comparable Dataset comprised 141 matched observations from six studies (Table S3).

Supplementary Methods S6. Predictive modelling and resampling specifications

Predictors for the primary and strict models were log10 administered mass concentration, log10 exposure duration, log10 primary diameter, a diameter-missing indicator, serum percentage, cell model, species, tissue group, raw assay label, assay family, surface group, charge group, protein-corona reporting, assay-interference control, data-source type, and size class. Numeric missing values were median-imputed within each training fold. Categorical values were imputed with the most frequent training-fold category and one-hot encoded with previously unseen test-fold categories ignored. Article_ID was used exclusively to define study groups for resampling and was never supplied as a model predictor. Preprocessing parameters were estimated from the corresponding training partitions and applied to held-out observations without refitting.

All learned models used inverse-study-frequency fitting weights so that articles contributing many observations did not dominate training. For article i containing n_i eligible observations, each observation received a fitting weight proportional to $1/n_i$. Benchmark evaluation used five-fold study-grouped out-of-fold predictions with shuffled group partitions and a fixed random seed of 42**.** The benchmark specifications were Ridge regression ($\alpha = 1.0$), Random Forest and Extra Trees (300 trees, minimum leaf size 3, maximum feature fraction 0.65), and CatBoost (300 iterations, depth 6, learning rate 0.05, RMSE loss). Stochastic models used random seed 42. The median dummy baseline used the training-fold median. Benchmark model selection prioritized study-balanced MAE, with observation-level performance considered as a complementary criterion.

The final nonlinear model used nested grouped cross-validation. Five outer GroupKFold partitions were used exclusively for performance estimation, and three inner grouped folds were used for hyperparameter selection within each outer-training set. Outer and inner partitions were shuffled with random seed 42 and Article_ID was the grouping variable. The random forest used 220 trees. Candidate

minimum-leaf-size / maximum-feature-fraction pairs were 2/0.50, 3/0.65, 5/0.80, and 8/1.00. Inner model selection minimized study-balanced MAE. Hyperparameter selection and preprocessing were confined to the outer-training data, and no observations from an outer held-out article were used during model selection. The selected hyperparameters and held-out performance for each outer fold are reported in Table S7.

The matched geometry-comparable analysis used leave-one-article-out validation. Ridge regularization was selected within each training set using three-fold grouped cross-validation. A conservative random-forest comparator used the identical held-out articles. Each model specification included one log10-transformed administered-dose metric and the same contextual predictors: log10 exposure duration, species, tissue group, assay family, surface group, charge group, and data-source type. Primary particle diameter was not included as an additional predictor in the original matched comparison because it was already embedded mathematically in the geometry-derived dose metrics. Study-balanced MAE was the primary matched-set metric. Paired contrasts were defined as MAE(mass) - MAE(alternative), so positive values favored the alternative metric. The separate post-review analysis incorporating primary diameter and a mass–diameter interaction is described in Supplementary Methods S9.5, with results presented in Tables S17 and S18.

Supplementary Methods S7. Performance metrics and uncertainty

Observation-level MAE, RMSE, and R^2 were calculated from held-out predictions. MAE and RMSE were expressed in percentage points of the normalized viability-related response. Study-balanced MAE was calculated by first computing MAE within each held-out article and then averaging equally across articles. Study-mean R^2 was calculated from article-level mean observed and mean predicted responses. Uncertainty for the final nested models was estimated using 1,000 Article_ID-cluster bootstrap replicates of held-out prediction pairs. Within each bootstrap replicate, articles were sampled with replacement, and all corresponding observed–predicted pairs were retained together to preserve within-study dependence. Bootstrap resampling used the existing out-of-fold predictions without repeating model fitting or hyperparameter tuning. Matched geometry contrasts used 5,000 article-cluster bootstrap replicates. The resulting 95% confidence intervals reflect uncertainty across the contributing studies rather than prospective external validation. Given that only six independent studies contributed to the matched comparison, these intervals should be interpreted cautiously.

Held-out permutation importance was calculated only in the strict ≤100 nm analysis. Within each outer test fold, each predictor was permuted 10 times and the increase in MAE relative to the unpermuted fold was recorded. Replicates were averaged within fold and then averaged across the five outer folds. Thus, ΔMAE quantifies predictive dependence in held-out data and is not interpreted as a causal

effect. Full predictor-specific permutation results and across-fold standard deviations are reported in Table S8.

Supplementary Methods S8. Reproducibility and R5 correction provenance

The final analyses were executed in Python 3.13 using scikit-learn 1.8.0 and CatBoost 1.2.8. The R5 correction was additive to the frozen R4 extraction archive and did not alter previously extracted R4 observations. It corrected the source identity for PSNP-1165 (DOI 10.3390/microplastics5010031), adding one eligible study, three particle arms, six archive observations, three primary-training rows, and one strict ≤100 nm row. Consequently, final study and dataset counts changed from 97 to 98 eligible studies, 1,698/81 to 1,701/82 primary rows/studies, and 994/59 to 995/60 strict rows/studies.

The effects of the R5 correction on study inclusion, analytical dataset composition, and model outputs are documented in Table S12. The R5-corrected analysis-ready workbook and the corresponding benchmark, nested random-forest, and permutation-importance output files are identified in Table S14. Additional post-review analyses were conducted using datasets derived from the frozen R5 archive, without modifying its underlying extraction records. Their methods and results are documented separately in Supplementary Methods S9 and Tables S15–S22. Any subsequently verified source-level corrections would require separate version documentation and reassessment of the affected analytical outputs.

Supplementary Methods S9 – Post-review sensitivity analyses and source-figure audit

S9.1. Common-study nanoscale restriction

To examine whether the apparent improvement following restriction to ≤100 nm was attributable to the narrower particle-size domain or to differences in study composition, an additional sensitivity analysis was conducted using studies contributing observations both within and above the ≤100 nm threshold. This subset comprised 22 independent articles and 905 experimental observations from the frozen Primary Mass-Concentration Dataset.

Two random-forest training configurations were evaluated. The broad-domain configuration used all eligible particle-size observations from the 22 studies, whereas the nanoscale-only configuration was trained exclusively on observations with primary particle diameters ≤100 nm. Both configurations were evaluated on the same 447 held-out nanoscale observations, with Article_ID-grouped outer validation and the established nested modelling procedure preserved. Thus, the comparison held the contributing studies and evaluation observations constant while varying the particle-size domain available during model training. Paired performance differences were evaluated using Article_ID-cluster bootstrap resampling. This analysis assessed the dependence of predictive performance on study composition and did not establish a causal effect of particle size (Supplementary Table S15).

S9.2. Missing-diameter complete-case restriction

To evaluate the influence of missing primary particle diameter and its handling during model preprocessing, a complete-case sensitivity analysis was performed. The 72 observations without a numerical primary geometric diameter were excluded from the Primary Mass-Concentration Dataset, leaving 1,629 observations from 78 studies.

The nested Article_ID-grouped random-forest procedure was repeated using the remaining observations, retaining the established preprocessing, inverse-study-frequency fitting weights, hyperparameter search space, and model-selection criteria. Performance was compared descriptively with the primary analysis. Because the restriction altered the contributing observations and studies, it was not treated as a paired comparison on identical held-out data (Supplementary Table S16).

S9.3. Reduced-predictor sensitivity

An additional analysis examined whether partially overlapping categorical and continuous descriptors influenced the predictive results. The raw assay label (Assay_Raw) and particle-size class (Size_Class) were removed from the primary predictor set, while the harmonized assay family (Assay_Family), continuous log10-transformed primary particle diameter, and the remaining original predictors were retained.

The analysis included the same 1,701 observations from 82 studies as the full primary dataset. The nested study-grouped random-forest procedure, inverse-study-frequency fitting weights, preprocessing strategy, and hyperparameter selection criteria were retained. The resulting performance was compared with the full-predictor model to assess the sensitivity of the findings to these potentially redundant descriptors (Supplementary Table S16).

S9.4. Quality-domain restriction

A further sensitivity analysis examined the influence of study reporting and methodological quality on predictive performance. Reporting quality and methodological quality were evaluated as separate categorical domains informed by the SciRAPnano in vitro assessment framework. Rather than constructing a composite numerical quality score, the analysis was restricted to studies classified as High in both domains according to the existing quality assessments.

This restriction yielded 1,575 observations from 70 independent studies. The established nested Article_ID-grouped random-forest procedure was repeated within this subset, retaining the original

predictor structure, preprocessing approach, weighting scheme, and model-selection criteria. Performance differences were interpreted as sensitivity to the quality-restricted evidence domain rather than as a direct paired comparison with the full primary dataset (Supplementary Table S16).

S9.5. Post hoc Ridge interaction sensitivity and six-study heterogeneity

To evaluate whether primary particle diameter provided additional predictive information beyond nominal mass concentration, supplementary Ridge-regression models were examined within the geometry-comparable dataset of 141 observations from six independent studies. The analysis used leave-one-article-out (LOAO) outer validation, with Ridge regularization selected through article-grouped inner cross-validation.

Three mass-based specifications were evaluated: a mass-concentration baseline, a model incorporating both log10-transformed mass concentration and log10-transformed primary particle diameter, and a model additionally incorporating their interaction as the product of these two transformed variables. The contextual predictors were retained across specifications. Particle-number and geometric surface-area representations were also examined within this supplementary comparison.

Model performance was evaluated using observation-level MAE, RMSE, R^2, study-balanced MAE, and study-mean R^2. Study-balanced MAE assigned equal weight to each of the six held-out articles, independently of the number of experimental observations contributed by that article. Paired contrasts were calculated from article-specific held-out errors, with 95% confidence intervals estimated using Article_ID-cluster bootstrap resampling.

This additional analysis was conducted after peer review as a model-specification sensitivity assessment. Its estimates are distinct from those of the original matched dose-metric comparison reported in Table 4 of the main manuscript and should not be substituted for them. Overall results and article-specific prediction errors are provided in Supplementary Tables S17 and S18, respectively.

S9.6. Independent human source-figure re-extraction and audit

An additional quality-control assessment was conducted to examine agreement between the frozen extraction archive and numerical values independently recovered from source figures. A separate human researcher who had not participated in the original extraction examined 30 selected figure-derived experimental conditions from ten source articles, with three conditions selected per article.

The second researcher received the source DOI, relevant figure or panel, and experimental-condition identifiers but was blinded to the response values recorded in the original extraction archive. For each attempted reading, the researcher documented the estimated response, figure-axis calibration, extraction method, source location, confidence in the reading, and any difficulties in identifying the

corresponding experimental condition or plotted series. Conditions that could not be quantified reliably were recorded as unresolved rather than assigned estimated or imputed values.

Following completion of the independent assessment, the returned records were matched to the frozen R5 extraction archive using Observation_ID, Article_ID, exposure duration, and administered dose. Numerical values could be recovered independently for 23 conditions from eight articles, whereas seven conditions remained non-quantifiable. For the 23 readable comparisons, agreement was evaluated on the raw figure-response scale. The mean absolute difference was 1.43 percentage points, the median absolute difference was 0.90 percentage points, and RMSE was 2.45 percentage points. Twenty of the 23 comparisons (87.0%) differed from the archived values by no more than two percentage points.

These statistics describe agreement among the selected readable conditions and should not be interpreted as a reliability estimate for the complete figure-derived evidence base. The seven unresolved conditions were retained in the audit denominator and were not imputed.

One additional targeted quality-control observation (OBS-0652) was examined separately and excluded from the 30-condition assessment. Its independent reading was 97.9%, compared with 90.0% in the frozen R5 archive. This discrepancy, together with outstanding source-panel interpretation and response-normalization issues involving other observations, requires source-level adjudication. The frozen R5 dataset and corresponding predictive-model outputs have not been modified on the basis of these unresolved comparisons. The audit findings and outstanding quality-control records are summarized in Supplementary Table S19.

Supplementary Tables

Table S1. Evidence-identification and full-text workflow summary.

Stage	n	Definition
Database records identified	4,649	Combined PubMed/MEDLINE, Web of Science, and Scopus retrieval
Duplicate records removed	2,149	Records removed during bibliographic deduplication
Unique title/abstract screening master	2,500	Consolidated deduplicated screening set
Excluded after title/abstract screening	1,724	Records not advanced to full-text retrieval
Advanced to full-text retrieval	776	Full-text candidate pool
Full texts obtained and assessed	153	Resolved full-text assessments
Included studies	98	Final eligibility
Excluded at full text	55	Final exclusions
Reports not retrieved at workflow closure	623	Retrieval unsuccessful or unresolved at workflow closure
Particle arms in frozen archive	227	R5-corrected archive
Condition-level observations	2,443	R5-corrected archive

Note. Counts are aligned with the final manuscript and the R5-corrected extraction archive. Reports not retrieved were not classified as full-text eligibility exclusions. A complete database-to-inclusion flow is presented in Supplementary Table S20 and Figure 1 of the main manuscript.

Table S2. Operational eligibility framework.

Component	Operational definition
Population / biological system	Mammalian cultured-cell systems; established cell lines or primary cells; human or other mammalian origin.
Exposure	PS-based particles with primary particle diameter 1–1000 nm; pristine or surface-functionalized; commercial/engineered, laboratory-generated/degraded, or environmentally derived/weathered provided that a PS-specific exposure arm could be evaluated separately.
Comparator	Untreated or vehicle control used for normalization/QC; not entered as dose = 0 training data.
Outcome	Quantitative continuous viability-, survival-, or metabolic-activity response reported or reliably extractable as percent of control.
Key exclusions	Exclusively in vivo; non-mammalian systems; non-PS material without separable PS arm; PS used solely as substrate/scaffold/carrier/tracer/support; no compatible quantitative viability-related endpoint; LDH release alone without a compatible viability-related outcome.

Table S3. Final analytical datasets.

Dataset	Studies	Observations	Rule / purpose
Frozen extraction archive	98	2,443	All retained condition-level observations across 227 particle arms
Primary mass concentration	82	1,701	Non-zero mass dose, exposure >0, response available, training eligible
Strict ≤100 nm	60	995	Primary dataset restricted by locked strict nanoscale flag
Geometry comparable	6	141	Identical observations with mass, particle number, and geometric surface-area metrics
Secondary areal dose	1	32	Administered μg/cm² observations retained separately and not pooled with the primary mass-concentration dataset**.**

Note. The Secondary Areal-Dose Dataset comprised 32 observations from a single study (PSNP-0024) and was not used for independent study-grouped predictive modelling.

Table S4. Deterministic derived-variable mappings used in the analysis-ready dataset.

Derived field	Rule	Purpose
Assay_Family	Keyword-based grouping: MTT/MTS/WST/CCK/tetrazolium → Tetrazolium/WST; alamar/resazurin/PrestoBlue → Resazurin; CellTiter-Glo/ATP → ATP; Neutral Red → NRU; trypan/coulter/count/clonogenic → Direct count/survival; Live/Dead/viability stain/calcein/annexin/acridine/eosin/PI → Staining/flow; else Other.	Reduces assay-label fragmentation while preserving Assay_Raw.
Tissue_Group	Keyword grouping of raw tissue into Gastrointestinal, Respiratory, Immune/hematopoietic, Hepatic, Nervous system, Reproductive, Renal, Skin/connective, Vascular, Placental, Other.	Provides transparent low-cardinality tissue feature.
Surface_Group	amin/NH2 → Aminated; carbox/COOH → Carboxylated; unmodified/plain/non-functionalized → Unmodified; missing → Not reported; remainder → Other/labeled.	Provides transparent low-cardinality surface feature.
Size_Class	≤100 nm; 101–500 nm; 501–1000 nm; Unknown.	Categorical predictor in the primary and strict models, alongside continuous primary diameter and its missingness indicator; excluded in the reduced-predictor sensitivity analysis.
Charge_Group	positive/cationic → Positive; negative → Negative; neutral/uncharged → Neutral/near-neutral; missing/not reported → Not reported; remainder → Other.	Reduces surface-charge label fragmentation while preserving the raw label.

Note. Raw source labels were retained alongside harmonized variables; mappings reduced label fragmentation without replacing source-level information.

Table S5. Full study-grouped benchmark performance in the primary mass-concentration dataset.

Model	MAE	RMSE	R^2	Study-balanced MAE	Study-mean MAE	Study-mean R^2
Dummy median	17.16	27.45	-0.071	17.09	14.32	-0.400
Ridge	19.19	25.36	0.086	18.29	14.34	-0.131
RandomForest	15.18	22.24	0.297	15.42	11.78	0.084
ExtraTrees	16.98	24.36	0.157	16.32	12.77	-0.066
CatBoost	14.68	21.38	0.350	15.65	12.06	0.048

Note. All values are from held-out study-grouped predictions. Random forest was carried forward because model selection prioritized study-balanced absolute error while considering observation-level performance.

Table S6. Final nested grouped random-forest performance and article-cluster-bootstrap 95% confidence intervals.

Dataset	Obs.	Studies	MAE (95% CI)	RMSE (95% CI)	R^2 (95% CI)	Study-balanced MAE	Study-mean R^2
Primary mass-concentration	1701	82	15.36 (12.78-19.41)	22.46 (17.94-28.42)	0.283 (0.033-0.461)	15.37	0.095
Strict ≤100 nm	995	60	14.37 (12.30-17.07)	21.23 (18.48-24.67)	0.447 (0.281-0.556)	15.60	0.322

Table S7. Outer-fold performance and selected random-forest hyperparameters.

Dataset	Fold	Held-out studies	Held-out rows	Min leaf	Max features	MAE	RMSE	R^2	SB MAE	Study-mean R^2
Primary	1	17	630	2	0.50	18.52	26.99	0.008	16.06	-0.057
Primary	2	17	245	2	0.50	16.29	21.85	0.315	17.84	0.432
Primary	3	16	301	3	0.65	13.83	18.87	0.419	13.86	0.242
Primary	4	16	260	2	0.50	14.18	20.63	0.541	16.01	-0.806
Primary	5	16	265	2	0.50	9.85	15.59	0.239	12.90	-0.262
Strict ≤100 nm	1	12	121	2	0.50	12.77	18.78	0.667	13.22	0.304
Strict ≤100 nm	2	12	216	3	0.65	16.20	22.58	0.436	17.24	0.333
Strict ≤100 nm	3	12	251	2	0.50	14.22	23.29	0.333	19.27	0.370
Strict ≤100 nm	4	12	158	3	0.65	12.76	18.23	0.080	14.58	-0.440
Strict ≤100 nm	5	12	249	2	0.50	14.71	20.72	0.446	13.71	0.559

Note. Hyperparameters were selected solely within the corresponding outer-training set using three-fold grouped inner validation. Article_ID defined the grouping variable for both inner and outer validation. SB MAE denotes study-balanced mean absolute error. Min leaf represents the minimum number of samples per terminal leaf, whereas Max features represent the proportion of model input features considered at each split. MAE and RMSE are expressed in percentage points of normalized response.

Table S8. Full held-out permutation importance in the strict ≤100 nm nested model.

Rank	Predictor	Mean ΔMAE	Across-fold SD
1	log10_Dose	4.778	2.007
2	Charge_Group	4.059	3.122
3	log10_Diameter	2.465	1.454
4	Surface_Group	0.409	0.653
5	Assay_Raw	0.164	0.411
6	Protein_Corona_Reported	0.152	0.207
7	Species	0.151	0.214
8	Serum_Percent	0.105	0.066
9	Data_Source_Type	0.087	0.057
10	log10_Exposure	0.058	0.108
11	Tissue_Group	0.032	0.188
12	Diameter_Missing	0.000	0.000
13	Size_Class	0.000	0.000
14	Assay_Family	-0.008	0.122
15	Cell_Model	-0.023	0.105
16	Assay_Interference_Control	-0.081	0.129

Note. Each predictor was permuted 10 times within each outer test fold. Values were averaged within fold and then across five folds. Negative values indicate that permutation did not worsen held-out MAE on average. ΔMAE values are expressed in percentage points of normalized response, and across-fold SD represents the standard deviation of the five outer-fold mean permutation effects. These values describe predictive dependence and should not be interpreted as causal effects.

Table S9. Matched geometry-comparable dose-metric performance (141 observations, six studies).

Model	Dose representation	MAE	Study-balanced MAE	R^2
Ridge	Mass concentration	7.52	11.68	0.186
Ridge	Particle number	13.10	15.02	-0.575
Ridge	Geometric surface area	12.89	14.22	-0.404
Random forest	Mass concentration	8.37	10.30	0.091
Random forest	Particle number	7.82	9.80	0.129
Random forest	Geometric surface area	8.49	11.29	0.052

Note. All models were evaluated on the same 141 experimental observations from six independent studies using leave-one-article-out validation. Study-balanced MAE was calculated by assigning equal weight to each held-out article. Paired random-forest contrasts and their 95% article-cluster-bootstrap confidence intervals are reported in Table S10. The additional post-review Ridge interaction sensitivity analysis is reported separately in Tables S17 and S18.

Table S10. Paired study-balanced MAE contrasts in the matched geometry-comparable random-forest analysis.

Model	Contrast	ΔMAE	95% article-cluster-bootstrap CI	Interpretation
Random forest	Mass - particle number	+0.50	-1.87 to 2.87	Positive favors particle number
Random forest	Mass - geometric surface area	-0.99	-3.00 to 0.50	Positive favors surface area

Note. Confidence intervals used 5,000 article-cluster bootstrap replicates and include zero for both geometry-derived administered-dose metrics. Paired contrasts were defined as study-balanced MAE(mass) − study-balanced MAE(alternative). Positive values favor the alternative dose metric, whereas negative values favor mass concentration. Because the analysis included only six independent studies, the estimated differences and their confidence intervals should be interpreted cautiously.

Table S11. Exploratory robustness subsets.

Restriction	Observations	Studies	MAE	Study-balanced MAE	R^2	Study-mean R^2
Directly reported numerical outcomes only	60	19	20.68	18.89	-0.419	-0.455
Assay-interference-control reported	628	30	20.72	20.19	0.111	-0.736

Note. These are domain-restriction diagnostics; they are not paired performance comparisons with the full primary dataset. Both restrictions altered the number and composition of contributing studies. Accordingly, differences in predictive performance should not be attributed solely to data-source type or assay-interference-control reporting. The negative R^2 in the directly reported subset does not, by itself, establish that figure-derived observations were inaccurate.

Table S12. R5 post-freeze correction provenance.

Item	R4 value	R5 value	Reason	Source	Model consequence
Final included studies	97	98	PSNP-1165 source mismatch corrected	DOI 10.3390/microplastics5010031; uploaded PDF	Study count changes
Primary model	1698 rows (81 studies)	1701 rows (82 studies)	Three non-zero MTT conditions added	Fig. 6A	Primary benchmark/nested RF rerun required
Strict ≤100 nm	994 rows (59 studies)	995 rows (60 studies)	40 nm PS-COOH condition added	Fig. 6A	Strict nested RF and permutation importance rerun required

Note. R4 remained immutable. R5 was an additive audited correction centered on PSNP-1165. The correction added one eligible study, three particle arms, and six condition-level observations to the extraction archive. Of these, three observations entered the primary training dataset and one entered the strict ≤100 nm subset. The affected predictive analyses were rerun using the corrected R5 datasets. Previously archived R4 observations were not modified.

Supplementary S1. Full search syntax

The following syntax records the final PS-focused concepts used for evidence identification. Database syntax was translated to the indexing structure of each platform. No machine-learning or geometry-aware dosimetry terms were required for retrieval. Eligibility decisions were made during screening rather than by analytical-model terms.

PubMed/MEDLINE

((("Polystyrenes"[Mesh] OR polystyrene[tiab]) AND ("Nanoparticles"[Mesh] OR "Microplastics"[Mesh] OR "Microspheres"[Mesh] OR nanoparticle*[tiab] OR nanoplastic*[tiab] OR microplastic*[tiab] OR nanospher*[tiab] OR nanobead*[tiab] OR nanoscal*[tiab] OR nanosized[tiab] OR "nano-sized"[tiab] OR "nano plastic"[tiab] OR "nano plastics"[tiab] OR submicron[tiab] OR "sub-micron"[tiab] OR ultrafine[tiab] OR latex[tiab] OR bead*[tiab] OR microspher*[tiab] OR microparticl*[tiab])) OR nanopolystyrene[tiab] OR "nano-polystyrene"[tiab] OR PSNP[tiab] OR PSNPs[tiab] OR "PS-NP"[tiab] OR "PS-NPs"[tiab] OR PSNPL[tiab] OR PSNPLs[tiab] OR "PS-NPL"[tiab] OR "PS-NPLs"[tiab]) AND ("Cell Survival"[Mesh] OR "Cell Death"[Mesh] OR "Cell Proliferation"[Mesh] OR cytotox*[tiab] OR viabil*[tiab] OR toxic*[tiab] OR "cell survival"[tiab] OR "cell death"[tiab] OR proliferat*[tiab] OR apopto*[tiab] OR necros*[tiab] OR "metabolic activity"[tiab] OR "membrane integrity"[tiab] OR MTT[tiab] OR MTS[tiab] OR XTT[tiab] OR "WST-1"[tiab] OR "WST-8"[tiab] OR "CCK-8"[tiab] OR CCK8[tiab] OR resazurin[tiab] OR "alamar blue"[tiab] OR "neutral red"[tiab] OR "trypan blue"[tiab] OR LDH[tiab] OR "lactate dehydrogenase"[tiab] OR "live/dead"[tiab] OR CellTiter*[tiab] OR ("in vitro"[tiab] AND cell*[tiab]))

Web of Science Core Collection - branch 1 (modern PS-nanoplastic; n = 1,310)

TS=(((polystyrene* OR polystyrol OR nanopolystyrene* OR "nano-polystyrene" OR "nano polystyrene") AND (nanoparticle* OR nanoplastic* OR microplastic* OR mesoplastic* OR "plastic nanoparticle" OR "plastic nanoparticles" OR "plastic microparticle" OR "plastic microparticles" OR nanospher* OR nanobead* OR nanoscal* OR nanosized OR "nano-sized" OR submicron OR "sub-micron")) AND (cytotox* OR viabil* OR "cell survival" OR "metabolic activity" OR MTT OR MTS OR XTT OR "WST-1" OR "WST-8" OR "CCK-8" OR CCK8 OR resazurin OR "alamar blue" OR "neutral red" OR "trypan blue" OR CellTiter* OR "live/dead"))

Web of Science Core Collection - branch 2 (legacy latex/bead/microsphere; n = 334)

TS=(((polystyrene* OR polystyrol) AND (latex OR bead* OR microspher*)) AND (cytotox* OR viabil* OR "cell survival" OR "metabolic activity" OR MTT OR MTS OR XTT OR "WST-1" OR "WST-8" OR "CCK-8" OR CCK8 OR resazurin OR "alamar blue" OR "neutral red" OR "trypan blue" OR CellTiter*))

Web of Science Core Collection - branch 3 (abbreviation safety net; n = 267)

TS=((PSNP OR PSNPs OR "PS-NP" OR "PS-NPs" OR PSNPL OR PSNPLs OR "PS-NPL" OR "PS-NPLs") AND (cytotox* OR viabil* OR "cell survival" OR "metabolic activity" OR MTT OR MTS OR XTT OR "WST-1" OR "WST-8" OR "CCK-8" OR CCK8 OR resazurin OR "alamar blue" OR "neutral red" OR "trypan blue" OR CellTiter* OR "live/dead"))

Web of Science audit combinations: #1 OR #2 = 1,503; #3 NOT (#1 OR #2) = 26; #1 OR #2 OR #3 = 1,529.

Scopus (TITLE-ABS-KEY translation of the final PS/viability concept structure)

TITLE-ABS-KEY((((polystyrene*) AND (nanoparticle* OR nanoplastic* OR microplastic* OR nanospher* OR nanobead* OR nanoscal* OR nanosized OR "nano-sized" OR "nano plastic" OR "nano plastics" OR submicron OR "sub-micron" OR ultrafine OR latex OR bead* OR microspher* OR microparticl* OR mesoplastic* OR "plastic microparticle*" OR "plastic nanoparticle*")) OR nanopolystyrene OR "nano-polystyrene" OR PSNP OR PSNPs OR "PS-NP" OR "PS-NPs" OR PSNPL OR PSNPLs OR "PS-NPL" OR "PS-NPLs") AND (cytotox* OR viabil* OR toxic* OR "cell survival" OR "cell death" OR proliferat* OR apopto* OR necros* OR "metabolic activity" OR "membrane integrity" OR MTT OR MTS OR XTT OR "WST-1" OR "WST-8" OR "CCK-8" OR CCK8 OR resazurin OR "alamar blue" OR "neutral red" OR "trypan blue" OR LDH OR "lactate dehydrogenase" OR "live/dead" OR CellTiter* OR ("in vitro" AND cell*)))

Search implementation note: the supplementary syntax is presented without using publication type, language, year, or open-access status as a scientific eligibility restriction, in line with the final manuscript. Database-interface exports and the screening archive were consolidated before eligibility adjudication.

Supplementary S2. Included-study summary

The table below summarizes the 98 final included studies in the R5-corrected analysis-ready archive. Evidence observations refer to all retained archive-level condition records for the study; primary and strict counts refer specifically to model-training rows in the corresponding analytical datasets.

Table S13. Characteristics of the final included studies.

Article ID	Year	Journal	DOI	Title	Archive obs.	Primary rows	≤100 nm rows	Dose min	Dose max	Time min (h)	Time max (h)	Median response (%)
PSNP-0003	2023	Part Fibre Toxicol	10.1186/s12989-023-00556-4	Polystyrene nanoplastic exposure induces excessive mitophagy by activating AMPK/ULK1 pathway in differentiated SH-SY5Y cells and dopaminergic neurons in vivo	5	4	4	0.5	500	48	48	90.5
PSNP-0005	2024	J Hazard Mater	10.1016/j.jhazmat.2024.134253	Effect of micro- and nanoplastic particles on human macrophages	7	6	0	37.5	1500	24	24	104.5
PSNP-0012	2022	Nat Commun	10.1038/s41467-022-32532-5	Anionic nanoplastic exposure induces endothelial leakiness	9	6	6	50	500	1	6	91
PSNP-0013	2025	Biol Res	10.1186/s40659-025-00629-y	Exposure to polystyrene	24	20	20	50	1200	3	48	96.4

Article ID	Year	Journal	DOI	Title	Archive obs.	Primary rows	≤100 nm rows	Dose min	Dose max	Time min (h)	Time max (h)	Median response (%)
				nanoparticles leads to dysfunction in DNA repair mechanisms in Caco-2 cells								
PSNP-0016	2022	Environ Pollut	10.1016/j.envpol.2022.119126	Polystyrene microplastic particles: In vivo and in vitro ocular surface toxicity assessment	5	4	4	5	100	48	48	68.5
PSNP-0017	2025	Part Fibre Toxicol	10.1186/s12989-025-00633-w	Polystyrene nanoplastics exposure induces cognitive impairment in mice via induction of oxidative stress and ERK/MAPK-mediated neuronal cuproptosis	4	3	3	250	750	24	24	90
PSNP-0024	2024	Small	10.1002/smll.202302907	A Multisystemic Approach Revealed Aminated	40	0	0					

Article ID	Year	Journal	DOI	Title	Archive obs.	Primary rows	≤100 nm rows	Dose min	Dose max	Time min (h)	Time max (h)	Median response (%)
				Polystyrene Nanoparticles-Induced Neurotoxicity								
PSNP-0025	2025	Int J Nanomedicine	10.2147/ijn.s567996	Quantifying Nanoplastic Toxicity Using Gold-Core Polystyrene Nanoparticles: In vivo Evaluation and Human Risk Extrapolation	12	8	8	1	10	12	24	94.445
PSNP-0026	2026	J Transl Med	10.1186/s12967-025-07508-w	Ferroptosis inhibition via the ROS-GPX4 axis drives microplastic-induced malignant progression of nasopharyngeal carcinoma	32	0	0					
PSNP-0029	2025	Adv Mater	10.1002/adma.202413413	Nanoplastics: Immune Impact, Detection, and	13	12	8	12.5	100	24	24	100

Article ID	Year	Journal	DOI	Title	Archive obs.	Primary rows	≤100 nm rows	Dose min	Dose max	Time min (h)	Time max (h)	Median response (%)
				Internalization after Human Blood Exposure by Single-Cell Mass Cytometry								
PSNP-0039	2022	Toxicol In Vitro	10.1016/j.tiv.2022.105314	Microplastics and nanoplastics: Size, surface and dispersant - What causes the effect?	59	0	0					
PSNP-0042	2023	J Appl Toxicol	10.1002/jat.4378	Internalization and cytotoxicity of polystyrene microplastics in human umbilical vein endothelial cells	12	10	0	5	100	24	48	98.5
PSNP-0044	2009	J Toxicol Sci	10.2131/jts.34.363	Cytotoxicity of nanoparticles independent from oxidative stress	6	5	5	50	500	24	24	4

Article ID	Year	Journal	DOI	Title	Archive obs.	Primary rows	≤100 nm rows	Dose min	Dose max	Time min (h)	Time max (h)	Median response (%)
PSNP-0048	2022	J Hazard Mater	10.1016/j.jhazmat.2022.129585	Bioaccumulation of differently-sized polystyrene nanoplastics by human lung and intestine cells	108	90	30	100	5000	4	24	111.35
PSNP-0056	2022	J Hazard Mater	10.1016/j.jhazmat.2022.128411	Effect of polystyrene nanoplastics and their degraded forms on stem cell fate	10	8	0	300	2400	24	24	96
PSNP-0058	2022	Environ Pollut	10.1016/j.envpol.2022.119924	The potential toxicity of polystyrene nanoplastics to human trophoblasts in vitro	12	9	9	10	100	24	72	107
PSNP-0064	2022	J Hazard Mater	10.1016/j.jhazmat.2022.128884	Microplastics exposure affects neural development of human pluripotent stem cell-derived cortical spheroids	4	3	0	5	100	624	624	64.3
PSNP-0066	2024	Int J Mol Sci	10.3390/ijms252011101	Bioaccumulation Rate of	12	0	0					

Article ID	Year	Journal	DOI	Title	Archive obs.	Primary rows	≤100 nm rows	Dose min	Dose max	Time min (h)	Time max (h)	Median response (%)
				Non-Biodegradable Polystyrene Microplastics in Human Epithelial Cell Lines								
PSNP-0069	2021	Toxicology	10.1016/j.tox.2021.152850	Polystyrene nanoplastics dysregulate lipid metabolism in murine macrophages in vitro	36	30	0	1	200	24	96	96.5
PSNP-0088	2021	Int J Mol Sci	10.3390/ijms22094406	Oxidative Properties of Polystyrene Nanoparticles with Different Diameters in Human Peripheral Blood Mononuclear Cells (In Vitro Study)	18	15	15	100	1000	24	24	91.3
PSNP-0091	2024	Sci Total Environ	10.1016/j.scitotenv.2023.169228	Polystyrene micro- and nanoplastics induce gastric toxicity through ROS	24	20	10	10	100	24	48	59.5

Article ID	Year	Journal	DOI	Title	Archive obs.	Primary rows	≤100 nm rows	Dose min	Dose max	Time min (h)	Time max (h)	Median response (%)
				mediated oxidative stress and P62/Keap1/Nrf2 pathway								
PSNP-0092	2022	J Hazard Mater	10.1016/j.jhazmat.2021.127508	In vitro study on the toxicity of nanoplastics with different charges to murine splenic lymphocytes	28	24	24	5	800	12	12	74
PSNP-0095	2023	Toxicology	10.1016/j.tox.2022.153385	Cellular response of THP-1 macrophages to polystyrene microplastics exposure	40	32	0	50	500	24	96	75
PSNP-0097	2025	J Transl Med	10.1186/s12967-025-06634-9	Polystyrene nanoplastics trigger pyroptosis in dopaminergic neurons through TSC2/TFEB-mediated disruption of autophagosome-lysosome fusion in	12	10	10	7.5	120	3	72	60

Article ID	Year	Journal	DOI	Title	Archive obs.	Primary rows	≤100 nm rows	Dose min	Dose max	Time min (h)	Time max (h)	Median response (%)
				Parkinson's disease								
PSNP-0102	2026	Cell Biol Toxicol	10.1007/s10565-025-10135-2	Nanoplastic toxicity and uptake in kidney cells: differential effects of concentration, particle size, and polymer type	20	16	12	0.2	200	24	24	100
PSNP-0109	2025	Int J Mol Sci	10.3390/ijms26104783	Uptake and Toxicity of Polystyrene NPs in Three Human Cell Lines	108	90	60	5	100	24	48	98.5
PSNP-0110	2025	Sci Rep	10.1038/s41598-025-19064-w	Polystyrene microplastics are internalized by human gingival fibroblasts, enhance cell motility and induce molecular changes revealed through proteomic analysis	18	15	0	0.25	50	24	72	94

Article ID	Year	Journal	DOI	Title	Archive obs.	Primary rows	≤100 nm rows	Dose min	Dose max	Time min (h)	Time max (h)	Median response (%)
PSNP-0116	2024	Front Public Health	10.3389/fpubh.2024.1385387	Polystyrene nanoplastics mediate oxidative stress, senescence, and apoptosis in a human alveolar epithelial cell line	21	18	0	10	500	24	96	82
PSNP-0122	2021	PLoS One	10.1371/journal.pone.0255120	Genotoxicity and oxidative stress induction by polystyrene nanoparticles in the colorectal cancer cell line HCT116	12	9	9	400	1200	4	48	98
PSNP-0129	2022	Int J Nanomedicine	10.2147/ijn.s381776	Cytotoxicity and Genotoxicity of Polystyrene Micro- and Nanoplastics with Different Size and Surface Modification in A549 Cells	36	32	32	2.5	400	24	24	85.95

Article ID	Year	Journal	DOI	Title	Archive obs.	Primary rows	≤100 nm rows	Dose min	Dose max	Time min (h)	Time max (h)	Median response (%)
PSNP-0137	2025	Int J Mol Sci	10.3390/ijms262311273	Polystyrene Micro- and Nanoplastic Exposure Triggers an Activation and Stress Response in Human Astrocytes	6	4	2	1	5	48	48	91
PSNP-0158	2022	Chem Biol Interact	10.1016/j.cbi.2022.110169	Fluoride enhances polystyrene nanoparticles cytotoxicity in colonocytes in vitro model	19	14	14	50	500	24	72	97.5
PSNP-0164	2014	Cell Biol Toxicol	10.1007/s10565-013-9265-y	Nano-sized and micro-sized polystyrene particles affect phagocyte function	94	78	36	25	500	4	24	90
PSNP-0166	2022	Environ Health Perspect	10.1289/ehp10873	Uptake, Transport, and Toxicity of Pristine and Weathered Micro- and Nanoplastics	60	48	16	0.1	100	24	24	103

Article ID	Year	Journal	DOI	Title	Archive obs.	Primary rows	≤100 nm rows	Dose min	Dose max	Time min (h)	Time max (h)	Median response (%)
				in Human Placenta Cells								
PSNP-0176	2021	PLoS One	10.1371/journal.pone.0260803	Uptake and toxicity of polystyrene micro/nanoplastics in gastric cells: Effects of particle size and surface functionalization	375	300	120	0.1	100	1	24	102.2
PSNP-0203	2012	Part Fibre Toxicol	10.1186/1743-8977-9-26	Action of polystyrene nanoparticles of different sizes on lysosomal function and integrity	25	20	20	10	100	24	120	94.5
PSNP-0205	2024	Sci Total Environ	10.1016/j.scitotenv.2024.173236	Hazard assessment of nanoplastics is driven by their surface-functionalization. Effects in human-derived primary endothelial cells	24	12	12	100	100	0.333	24	79.78

Article ID	Year	Journal	DOI	Title	Archive obs.	Primary rows	≤100 nm rows	Dose min	Dose max	Time min (h)	Time max (h)	Median response (%)
PSNP-0207	2026	Arch Toxicol	10.1007/s00204-026-04417-9	Real-life nanoplastics induce endothelial dysfunction in primary human endothelial cells	2	1	0	25	25	24	24	102
PSNP-0255	2023	PLoS One	10.1371/journal.pone.0289473	Correlation between cellular uptake and cytotoxicity of polystyrene micro/nanoplastics in HeLa cells: A size-dependent matter	54	48	40	1	500	4	4	97
PSNP-0262	2023	Environ Sci Technol	10.1021/acs.est.3c01134	Role of Residual Monomers in the Manifestation of (Cyto)toxicity by Polystyrene Microplastic Model Particles	28	28	0	0.1	1900	24	24	85.5

Article ID	Year	Journal	DOI	Title	Archive obs.	Primary rows	≤100 nm rows	Dose min	Dose max	Time min (h)	Time max (h)	Median response (%)
PSNP-0291	2021	Biomolecules	10.3390/biom11101442	Long-Term Effects of Polystyrene Nanoplastics in Human Intestinal Caco-2 Cells	10	0	0					
PSNP-0339	2021	Part Fibre Toxicol	10.1186/s12989-021-00428-9	Prospects on the nano-plastic particles internalization and induction of cellular response in human keratinocytes	36	30	30	25	500	24	144	105
PSNP-0370	2014	Toxicol Appl Pharmacol	10.1016/j.taap.2014.07.017	Use of whole genome expression analysis in the toxicity screening of nanoparticles	24	18	9	50	200	24	24	91
PSNP-0376	2010	J R Soc Interface	10.1098/rsif.2010.0068.focus	Biomechanical effects of environmental and engineered particles on human airway smooth muscle cells	5	4	4	25	200	24	24	97

Article ID	Year	Journal	DOI	Title	Archive obs.	Primary rows	≤100 nm rows	Dose min	Dose max	Time min (h)	Time max (h)	Median response (%)
PSNP-0387	2023	Part Fibre Toxicol	10.1186/s12989-023-00513-1	Short- and long-term polystyrene nano- and microplastic exposure promotes oxidative stress and divergently affects skin cell architecture and Wnt/beta-catenin signaling	16	8	0	100	100	168	672	92
PSNP-0397	2015	PLoS One	10.1371/journal.pone.0123297	Specific uptake and genotoxicity induced by polystyrene nanobeads with distinct surface chemistry on human lung epithelial cells and macrophages	120	0	0					
PSNP-0432	2025	Sci Rep	10.1038/s41598-025-91248-w	Primary astrocytes as a cellular depot of	60	48	48	0.5	50	6	72	94.5

Article ID	Year	Journal	DOI	Title	Archive obs.	Primary rows	≤100 nm rows	Dose min	Dose max	Time min (h)	Time max (h)	Median response (%)
				polystyrene nanoparticles								
PSNP-0478	2013	PLoS One	10.1371/journal.pone.0056791	Assessment of long-term effects of nanoparticles in a microcarrier cell culture system	24	20	10	50	500	24	24	80
PSNP-0719	2024	Heliyon	10.1016/j.heliyon.2024.e30518	Cytotoxicity of amine-modified polystyrene MPs and NPs on neural stem cells cultured from mouse subventricular zone	8	8	4	20	100	24	24	25
PSNP-0723	2023	Toxics	10.3390/toxics11070627	Evaluation of In Vitro Genotoxicity of Polystyrene Nanoparticles in Human Peripheral Blood Mononuclear Cells	20	18	0	15	100	3	24	95.5
PSNP-0744	2026	Toxics	10.3390/toxics14060507	The Particle Size Effect: Cytotoxicity	81	72	48	12.5	2000	24	72	97

Article ID	Year	Journal	DOI	Title	Archive obs.	Primary rows	≤100 nm rows	Dose min	Dose max	Time min (h)	Time max (h)	Median response (%)
				and Cellular Uptake of Polystyrene Nanoplastics in Human Keratinocytes								
PSNP-0908	2020	RSC Adv	10.1039/c9ra09752j	Biocompatible UV-absorbing polymer nanoparticles prepared by electron irradiation for application in sunscreen	16	0	0					
PSNP-1207	2025	Microplastics and Nanoplastics	10.1186/s43591-025-00170-5	Functionalized polystyrene nanoplastics induce distinct toxicity and transcriptomic changes in human intestinal Caco-2 cells	99	90	90	1	100	24	96	96
PSNP-1253	2025	Journal of Nanobiotechnology	10.1186/s12951-025-03545-1	Molecular mechanism differences between nanoplastics and	18	15	5	1000	5000	24	24	51

Article ID	Year	Journal	DOI	Title	Archive obs.	Primary rows	≤100 nm rows	Dose min	Dose max	Time min (h)	Time max (h)	Median response (%)
				microplastics in colon toxicity: nanoplastics induce ferroptosis-mediated immunogenic cell death, while microplastics cause cell metabolic reprogramming								
PSNP-1408	2026	NANOMATERIALS	10.3390/nano16020105	Polystyrene Nanoplastics Increase Macrophage Bactericidal Activity Through a Mechanism Involving Reactive Oxygen Species and Itaconate	6	5	5	1	10	24	24	93
PSNP-1412	2025	Toxics	10.3390/toxics13121060	Toxic Effects of Neonicotinoid Insecticide Imidacloprid and Polystyrene	8	7	0	150	1050	48	48	55

Article ID	Year	Journal	DOI	Title	Archive obs.	Primary rows	≤100 nm rows	Dose min	Dose max	Time min (h)	Time max (h)	Median response (%)
				Microplastics on Rat Neuroblastoma B104 Cells								
PSNP-1421	2025	Toxics	10.3390/toxics13070605	Impact of Polystyrene Microplastics on Human Sperm Functionality: An In Vitro Study of Cytotoxicity, Genotoxicity and Fertility-Related Genes Expression	9	6	0	105	210	0.5	1.5	68.8
PSNP-1454	2024	International Journal of Bioprinting	10.36922/ijb.1403	Distinct toxicity of microplastics/TBBPA co-exposure to bioprinted liver organoids derived from hiPSCs of healthy and patient donors	2	1	0	0.6	0.6	72	72	98
PSNP-1470	2023	Chinese Journal of	10.1016/j.cjac.2022.100149	Novel quartz crystal microbalance	10	5	2	100	100	24	24	78

Article ID	Year	Journal	DOI	Title	Archive obs.	Primary rows	≤100 nm rows	Dose min	Dose max	Time min (h)	Time max (h)	Median response (%)
		Analytical Chemistry		cytosensor for real-time monitoring of polystyrene nanospheres effect on cell apoptosis								
PSNP-1538	2018	NANOMATERIALS	10.3390/nano8121028	Surface Charge-Dependent Cellular Uptake of Polystyrene Nanoparticles	28	14	14	50	50	4	4	96
PSNP-1590	2014	PLoS ONE	10.1371/journal.pone.0108025	High content analysis provides mechanistic insights on the pathways of toxicity induced by amine-modified polystyrene nanoparticles	24	20	20	1.5	100	24	24	95.5
PSNP-1667	2025	Ecotoxicology and Environmental Safety	10.1016/j.ecoenv.2025.117712	Cardiotoxicity of polystyrene nanoplastics and associated mechanism	14	12	12	200	300	24	48	82.5

Article ID	Year	Journal	DOI	Title	Archive obs.	Primary rows	≤100 nm rows	Dose min	Dose max	Time min (h)	Time max (h)	Median response (%)
				of myocardial cell injury in mice								
PSNP-1679	2025	PLOS ONE	10.1371/journal.pone.0337568	Exposure to polystyrene nanoparticles induce disruption of mitochondrial homeostasis and impairs trophoblast cell invasion and migration via MDM2/ROCK1 pathway	4	2	0	200	200	24	24	64.35
PSNP-1681	2025	Journal of Nanobiotechnology	10.1186/s12951-025-03747-7	Plastic tableware use, microplastic accumulation, and sperm quality: from epidemiological evidence to FOXA1/p38 mechanistic insights	7	6	6	1	200	24	24	81
PSNP-1718	2025	Science of the Total	10.1016/j.scitotenv.2025.180883	FGFR2-regulated cytoskeletal rearrangeme	22	20	0	0.1	500	24	48	82

Article ID	Year	Journal	DOI	Title	Archive obs.	Primary rows	≤100 nm rows	Dose min	Dose max	Time min (h)	Time max (h)	Median response (%)
		Environment		nt disturbs autophagy flux induced by polystyrene nanoplastics								
PSNP-1733	2026	Cell Biology and Toxicology	10.1007/s10565-026-10152-9	Polystyrene Nanoplastics induced placental toxicology by activating Keap1-mediated ferroptosis via METTL3-dependent m6A methylation	6	5	5	6.25	100	24	24	80
PSNP-1735	2025	International Journal of Toxicology	10.1177/10915818241305086	Exploring the Biological Effects of Polystyrene Nanoplastics on Spermatogenesis: Insights From Transcriptomic Analysis in Mouse Spermatocytes	4	3	3	5	25	24	24	103

Article ID	Year	Journal	DOI	Title	Archive obs.	Primary rows	≤100 nm rows	Dose min	Dose max	Time min (h)	Time max (h)	Median response (%)
PSNP-1739	2025	Infection	10.1007/s15010-025-02565-1	Micro- and nanoplastics reduce the phagocytosis and intracellular killing of E. coli by THP1-Blue™ NFκB monocytes	24	20	0	1	500	24	216	96
PSNP-1746	2025	Journal of Translational Medicine	10.1186/s12967-025-06868-7	Polystyrene microspheres could inhibit the ferroptosis of prostate cancer cells via USP39/IGF2BP3/MAPK4 axis	8	6	0	200	1000	12	12	136.5
PSNP-1791	2025	Archives of Toxicology	10.1007/s00204-025-04046-8	Microplastics as benzo-a-pyrene carriers: genotoxicity assessment simulating human gastric digestion	0	0	0					
PSNP-1799	2026	Particle and	10.1186/s12989-026-00661-0	Supplemental exposure to	2	1	0	100	100	24	24	98.8

Article ID	Year	Journal	DOI	Title	Archive obs.	Primary rows	≤100 nm rows	Dose min	Dose max	Time min (h)	Time max (h)	Median response (%)
		Fibre Toxicology		polystyrene nanoplastics synergistically amplifies calcium oxalate crystal–induced injury to renal tubular epithelium, accelerating the formation of calcium oxalate kidney stones								
PSNP-1827	2025	Journal of Nanobiotechnology	10.1186/s12951-025-03620-7	Internalized polystyrene nanoplastics trigger testicular damage and promote ferroptosis via CISD1 downregulation in mouse spermatocyte	12	9	9	50	200	6	24	94.6
PSNP-1837	2025	Environmental Science and Technology	10.1021/acs.est.5c00380	Polystyrene Micro(nano)plastic-Induced Ca2+Disorder Activates Endoplasmic	24	24	16	10	5000	24	24	68.5

Article ID	Year	Journal	DOI	Title	Archive obs.	Primary rows	≤100 nm rows	Dose min	Dose max	Time min (h)	Time max (h)	Median response (%)
				Reticulum and Mitochondrial Signaling Pathways to Synergistically Promote Apoptosis in Human Lung Cells								
PSNP-1842	2025	Journal of Nanobiotechnology	10.1186/s12951-025-03535-3	Baicalin targets YTHDC2 and alleviates male reproductive toxicity caused by co-exposure to nanoplastics and manganese through m6A-dependent pathway	7	0	0					
PSNP-1854	2024	Toxicology Research	10.1093/toxres/tfae188	Proteomics reveals that nanoplastics with different sizes induce hepatocyte apoptosis in mice through	10	8	4	5	100	48	48	98.5

Article ID	Year	Journal	DOI	Title	Archive obs.	Primary rows	≤100 nm rows	Dose min	Dose max	Time min (h)	Time max (h)	Median response (%)
				distinct mechanisms involving mitophagy dysregulation and cell cycle arrest								
PSNP-1870	2024	Ecotoxicology and Environmental Safety	10.1016/j.ecoenv.2024.116439	Exposure to polystyrene nanoplastics induces hepatotoxicity involving NRF2-NLRP3 signaling pathway in mice	4	3	3	50	150	24	24	76
PSNP-1876	2024	Environmental Toxicology and Pharmacology	10.1016/j.etap.2024.104563	Lack of effects of polystyrene micro- and nanoplastics on activity and expression of human drug transporters	8	6	6	1	100	48	48	103
PSNP-1882	2024	Journal of Zhejiang University: Science B	10.1631/jzus.b2300138	Nrf2-mediated ferroptosis of spermatogenic cells involved in male	14	12	12	12.5	400	24	24	85

Article ID	Year	Journal	DOI	Title	Archive obs.	Primary rows	≤100 nm rows	Dose min	Dose max	Time min (h)	Time max (h)	Median response (%)
				reproductive toxicity induced by polystyrene nanoplastics in mice; [Nrf2 介导的生精细胞铁死亡参与了聚苯乙烯纳米塑料导致的小鼠雄性生殖毒性]								
PSNP-1884	2022	Journal of Hazardous Materials	10.1016/j.jhazmat.2022.129470	Long-term exposure to nanoplastics alters molecular and functional traits related to the carcinogenic process	5	4	4	10	100	24	24	87
PSNP-1889	2024	Frontiers in Molecular Neuroscience	10.3389/fnmol.2024.1345536	Ototoxicity of polystyrene nanoplastics in mice, HEI-OC1 cells and zebrafish	5	4	4	50	500	24	24	65.5

Article ID	Year	Journal	DOI	Title	Archive obs.	Primary rows	≤100 nm rows	Dose min	Dose max	Time min (h)	Time max (h)	Median response (%)
PSNP-1896	2024	Nature Nanotechnology	10.1038/s41565-024-01683-5	Polystyrene nanoparticles trigger aberrant condensation of TDP-43 and amyotrophic lateral sclerosis-like symptoms	4	0	0					
PSNP-1901	2023	Toxics	10.3390/toxics11050441	Distribution of Micro-Nano PS, DEHP, and/or MEHP in Mice and Nerve Cell Models In Vitro after Exposure to Micro-Nano PS and DEHP	14	12	12	25	800	24	24	89
PSNP-1903	2024	Archives of Environmental Contamination and Toxicology	10.1007/s00244-024-01073-x	UVB-Aged Microplastics and Cellular Damage: An in Vitro Study	0	0	0					

Article ID	Year	Journal	DOI	Title	Archive obs.	Primary rows	≤100 nm rows	Dose min	Dose max	Time min (h)	Time max (h)	Median response (%)
PSNP-1907	2025	Journal of Army Medical University	10.16016/j.2097-0927.202502043	Effect of polystyrene micro/nanoplastics on mesenchymal phenotypic transformation in testicular Sertoli cells; [聚苯乙烯微/纳米塑料对睾丸支持细胞间质表型转化的影响]	0	0	0					
PSNP-1908	2024	Chemosphere	10.1016/j.chemosphere.2024.141463	Microplastics role in cell migration and distribution during cancer cell division	0	0	0					
PSNP-1913	2022	International Journal of Molecular Sciences	10.3390/ijms23020806	Nanomechanical Atomic Force Microscopy to Probe Cellular Microplastics Uptake and Distribution	16	12	3	1	10	24	24	91.5
PSNP-1935	2024	Particle and Fibre	10.1186/s12989-024-00574-w	Exposure to high dose of polystyrene	9	7	7	50	500	24	48	79

Article ID	Year	Journal	DOI	Title	Archive obs.	Primary rows	≤100 nm rows	Dose min	Dose max	Time min (h)	Time max (h)	Median response (%)
		Toxicology		nanoplastics causes trophoblast cell apoptosis and induces miscarriage								
PSNP-1955	2025	Frontiers in Immunology	10.3389/fimmu.2025.1609208	Polystyrene nanoplastics induce oxidative stress in Aurelia coerulea polyps, microglia, and mice	8	7	7	10	600	12	12	69
PSNP-1985	2024	Particle and Fibre Toxicology	10.1186/s12989-024-00578-6	Acute exposure to polystyrene nanoparticles promotes liver injury by inducing mitochondrial ROS-dependent necroptosis and augmenting macrophage-hepatocyte crosstalk	9	8	7	10	100	1	4	87.5
PSNP-2104	2020	Scientific Reports	10.1038/s41598-020-77191-y	Immortalisation of primary	40	32	32	25	100	24	24	76

Article ID	Year	Journal	DOI	Title	Archive obs.	Primary rows	≤100 nm rows	Dose min	Dose max	Time min (h)	Time max (h)	Median response (%)
				human alveolar epithelial lung cells using a non-viral vector to study respiratory bioreactivity in vitro								
PSNP-2159	2019	Journal of Nanobiotechnology	10.1186/s12951-019-0495-x	Role of nanoparticle size and sialic acids in the distinct time-evolution profiles of nanoparticle uptake in hematopoietic progenitor cells and monocytes	12	6	4	50	50	1	4	102
PSNP-2189	2022	Chemosphere	10.1016/j.chemosphere.2022.134947	Polystyrene micro and nano-particles induce metabolic rewiring in normal human colon cells: A risk	5	4	0	1	20	48	48	94

Article ID	Year	Journal	DOI	Title	Archive obs.	Primary rows	≤100 nm rows	Dose min	Dose max	Time min (h)	Time max (h)	Median response (%)
				factor for human health								
PSNP-2276	2014	Lab on a Chip	10.1039/c4lc00371c	Body-on-a-chip simulation with gastrointestinal tract and liver tissues suggests that ingested nanoparticles have the potential to cause liver injury	8	0	0					
PSNP-2312	2015	Proteomics	10.1002/pmic.201300423	Cellular response to empty and palladium-conjugated amino-polystyrene nanospheres uptake: A proteomic study	8	4	0	86	86	48	48	101.65
PSNP-2317	2012	International Journal of Nanomedicine	10.2147/IJN.S26601	In vitro placental model optimization for nanoparticle transport studies	22	20	20	100	1000	24	24	107

Article ID	Year	Journal	DOI	Title	Archive obs.	Primary rows	≤100 nm rows	Dose min	Dose max	Time min (h)	Time max (h)	Median response (%)
PSNP-2414	2015	Journal of Biomedical Materials Research - Part A	10.1002/jbm.a.35169	Macrophage reprogramming: Influence of latex beads with various functional groups on macrophage phenotype and phagocytic uptake in vitro	36	0	0					
PSNP-2432	2012	International Journal of Nanomedicine	10.2147/ijn.s29334	Cellular entry of nanoparticles via serum sensitive clathrin-mediated endocytosis, and plasma membrane permeabilization	8	0	0					
PSNP-1165	2026	Microplastics	10.3390/microplastics5010031	The Role of the Size and Surface Chemistry of Polystyrene Micro- and Nanobeads in the Interaction	6	3	1	10	10	24	24	75

Article ID	Year	Journal	DOI	Title	Archive obs.	Primary rows	≤100 nm rows	Dose min	Dose max	Time min (h)	Time max (h)	Median response (%)
				with an Advanced In Vitro Tri-Culture Intestinal Barrier Model								

Note. Dose ranges refer to harmonized mass-concentration rows where available; blank cells indicate that the study did not contribute eligible mass-concentration model rows.

Supplementary data and reproducibility manifest

Table S14. Reproducibility files used to generate the final manuscript results.

File class	Filename	Contents
Analysis-ready workbook	PSNP_Analysis_Ready_v2_R5_CORRECTED.xlsx	Primary, strict, areal-dose, geometry-comparable, study-summary, codebook, mapping, and R5-provenance sheets.
Primary nested-model output	PSNP_R5_Primary_NestedRF_FINAL.json	Overall metrics, cluster-bootstrap CIs, fold-level metrics, selected hyperparameters, and out-of-fold predictions.
Strict nested-model output	PSNP_R5_Strict_NestedRF_FINAL.json	Strict-model metrics, CIs, fold-level results, selected hyperparameters, and out-of-fold predictions.
Strict permutation output	PSNP_R5_Strict_Permutation_FINAL.json	Full 16-predictor held-out permutation importance with fold-level values.
Benchmark output	PSNP_R5_Benchmark_FINAL.json	Five-model R5 study-grouped benchmark results.

Note. The files listed above identify the frozen R5 analysis-ready workbook and its associated model outputs. Listing these files does not establish that all source-level extractions have been independently verified. The post-review sensitivity analyses and source-figure audit are reported separately in Supplementary Methods S9 and Tables S15–S22.

Post-review sensitivity analyses and audit results

Table S15. Common-study nanoscale sensitivity on identical held-out ≤100 nm observations.

Training domain	Studies	Held-out nano rows	MAE	RMSE	R^2	Study-balanced MAE
Broad sizes in the same 22 studies	22	447	19.83	26.80	0.292	19.70
≤100 nm only in the same 22 studies	22	447	19.77	27.13	0.274	20.61

Note. Paired article-cluster bootstrap 95% confidence intervals for nano-only minus broad training were: MAE, −1.92 to 2.35 percentage points; R^2, −0.166 to 0.103; and study-balanced MAE, −0.26 to 2.11 percentage points. All intervals included zero.

Table S16. Primary and post-review robust-configuration performance.

Analysis domain	Rows	Studies	MAE	RMSE	R^2	Study-balanced MAE	Study-mean R^2
Full primary	1,701	82	15.36	22.46	0.283	15.37	0.095
Numerical diameter complete case	1,629	78	15.55	22.35	0.310	15.67	0.108
Reduced predictors	1,701	82	15.58	23.18	0.236	15.77	−0.006
Dual-high quality	1,575	70	15.42	22.51	0.270	15.55	−0.021

Note. Each restriction modifies the data domain or model covariates. Metrics are from independent study-grouped out-of-fold predictions; these are not within-row paired causal contrasts.

Table S17. Reviewer-requested post hoc Ridge interaction sensitivity.

Post hoc Ridge model	MAE	RMSE	R^2	Study-balanced MAE	Study-mean R^2
Mass baseline	7.932	11.884	0.180	10.976	−0.166
Mass + diameter	7.868	11.851	0.184	11.028	−0.200
Mass + diameter + interaction	7.335	11.705	0.204	10.428	−0.034
Particle number	13.141	17.041	−0.687	17.179	−2.827
Geometric surface area	9.154	13.016	0.016	13.331	−0.858

Note. Table S17 reports a separately executed post hoc leave-one-article-out Ridge sensitivity analysis, not a re-tabulation of the original matched-model predictions in Table S9 or main-text Table 4. Both analyses retain contextual predictors (log10 exposure duration, species, tissue group, assay family, surface group, charge group, and data-source type); the difference between the numerical results must not be attributed to absence of these predictors in Table S17. Within the post hoc analysis, the paired study-balanced mass-minus-interaction contrast was +0.55 percentage points (95% article-cluster-bootstrap CI, −0.73 to 2.13).

Table S18. Article-specific errors in the post hoc matched Ridge sensitivity.

Article_ID	n	MAE: mass	MAE: mass + diameter	MAE: mass + diameter + interaction	MAE: particle number	MAE: surface area	ΔMAE: mass − interaction	ΔMAE: mass − number	ΔMAE: mass − surface
PSNP-0003	4	1.99	1.98	1.51	7.09	5.96	0.48	−5.10	−3.97
PSNP-0013	20	9.68	9.53	10.18	9.44	9.89	−0.50	0.24	−0.20
PSNP-0109	90	5.31	5.21	4.39	11.32	6.36	0.93	−6.01	−1.04
PSNP-0122	9	11.58	11.47	13.25	15.02	13.06	−1.67	−3.43	−1.48
PSNP-1421	6	18.87	19.57	14.70	40.15	26.22	4.16	−21.29	−7.35
PSNP-1901	12	18.42	18.41	18.53	20.05	18.51	−0.11	−1.63	−0.09

Note. Positive paired mass-minus-alternative differences favor the alternative on that article. Study heterogeneity is substantial; the six Article_IDs are the independent inferential units.

Table S19. Blinded independent human figure re-extraction: agreement and outstanding QC.

Technical audit summary	Result
Study sampling frame	Ten source articles, three conditions per article (30 attempted readings)
Extractor	Separate human researcher, blinded to originally extracted outcome values
Independently readable numeric pairs	23 of 30 conditions, from 8 of 10 articles
Unresolved/non-quantifiable conditions	7 of 30; retained in audit denominator and not numerically imputed
Within ±2 percentage points (raw scale)	20 of 23 readable pairs (87.0%)
Mean absolute difference (raw scale)	1.43 percentage points across 23 readable pairs
Median absolute difference (raw scale)	0.90 percentage points across 23 readable pairs
RMSE (raw scale)	2.45 percentage points across 23 readable pairs
Separate targeted QC observation	OBS-0652: independent 97.9% versus R5 archived 90.0%; excluded from the 30-condition assessment
Other QC issues	Article_ID PSNP-0744 control normalization; OBS-1643 and source-panel ambiguity remain open
Frozen data/model status	R5 not overwritten; no corrected-model re-estimation performed

Note. Agreement statistics apply only to 23 readable conditions from a purposively selected set of 30 attempted re-extractions and do not quantify reliability for all 1,641 figure-derived primary observations. The seven unreadable conditions were not imputed. The separate targeted OBS-0652 discrepancy and the outstanding source-panel/normalization queries (including OBS-1643) are recorded transparently but have not been independently resolved. Therefore the frozen R5 values, models, and Table 4 aggregates were not altered. These QC findings limit the precision of claims from source-digitized responses and cannot be used to infer the prevalence of extraction errors across the corpus.

Table S20. Full evidence-identification and screening-flow audit.

Stage	n
PubMed records	991
Web of Science records	1,529
Scopus records	2,129
Database records identified	4,649
Duplicates removed	2,149
Unique titles/abstracts screened	2,500
Not advanced to full text	1,724
Full-text retrieval candidates	776
Full texts not retrieved	623
Full texts obtained and assessed	153
Full-text exclusions	55
Included studies	98

Note. Records not retrieved were coded as such in the archived Retrieval_Rescue_623 file. The archived workflow did not document defensible finer retrieval-failure reasons. Therefore, the 623 records cannot be assigned to mutually exclusive causal subcategories retrospectively.

Supplementary Table S21. Frozen R5 primary RF OOF predictions versus training-fold dummy predictors on the identical directly reported subset (60 observations, 19 studies).

Model / evaluation	n	MAE (pp)	RMSE (pp)	R^2	Study-balanced MAE (pp)
Frozen R5 primary RF OOF filtered to directly reported	60	18.199	22.869	0.135	17.392
Training-fold directly reported mean dummy	60	21.584	27.067	-0.212	20.684
Training-fold directly reported median dummy	60	20.228	26.940	-0.200	18.549

Note. Data_Source_Type = “Directly reported” was joined by Observation_ID to the archived 1,701-row R5 Primary_Model table; RF predictions and outer-fold assignments were taken unchanged from PSNP_R5_Primary_NestedRF_FINAL.json. For each of the original five outer folds, mean and median dummy estimates used the directly reported observations in the other four folds only (held-out responses were not used). No RF model was refitted and no source or outcome values were altered. The independently retrained direct-only restriction previously reported in Table S11 (R^2 −0.419) is a different analysis and remains unchanged. Across 5,000 paired article-cluster bootstrap replicates, the study-balanced MAE contrasts (dummy minus RF) were +3.29 percentage points (mean dummy; 95% CI −1.73 to 8.33) and +1.16 percentage points (median dummy; 95% CI −3.69 to 5.49). Both intervals included zero; inference is limited by 19 contributing studies.

Supplementary Table S22. Study-level MAE of the frozen R5 full-primary nested RF OOF predictions, restricted to the six geometry-comparable articles (141 observations).

Article_ID	Observations	Original R5 primary OOF fold	Primary RF OOF MAE (pp)
PSNP-0003	4	1	3.382
PSNP-0013	20	3	9.485
PSNP-0109	90	5	5.480
PSNP-0122	9	4	8.647
PSNP-1421	6	4	15.195
PSNP-1901	12	4	14.731

Critical scope note. Table S22 reports errors of the frozen PRIMARY nested random forest, trained within the original full-primary outer folds and evaluated only on the six geometry-comparable studies. It is not a decomposition of the three dose-metric-specific LOAO random forests reported in main-text Table 4. The per-observation predictions from the original Table 4 random forests were not retained in the archive; the matched 141-observation dataset and the published study-grouped analysis specification are documented (Methods 2.6; Supplementary Methods S4 and S9.5). Their study-by-study RF errors therefore cannot be recovered directly from the retained original prediction outputs. We have not substituted a re-execution for the original Table 4 estimates. Table S22 supplies a separately labelled descriptive primary-RF OOF diagnostic; metric-specific study-level post hoc Ridge results appear in Table S18.
